# Supplementary material for: Improving tomato yield and production economics with Nano-NPK fertilization and salicylic acid chemigation
Source: PLoS One. 2026 May 4;21(5):e0348207. doi: 10.1371/journal.pone.0348207 (PMC13138637; doi:10.1371/journal.pone.0348207)
Supplement: S2 File — (DOCX) [file pone.0348207.s002.docx]

**Supporting Information**

**S1 Table.** Economic, energy, and environmental assessment of processing tomato production with 120 kg NPK fertilization per ha (control treatment) in 2022 and 2023.

________________________________________________________________________________________________________

**Economics Quantity /ha Unit $/Unit Total ($/ha)**

**2022 2023 2022 2023**

Economic output 40.3 43.5 Ton $189.0 $7,616.7 $8,221.5

Economic input

Fertilization

Pre-plant fertilization (Triple 19 NPK) 105 105 kg $0.6 $59.0 $59.0

Drip fertilization (Triple 20 NPK) 500 500 kg $1.6 $800.0 $800.0

Nanoliquid - NanoPro 300 300 mL $0.0 $0.0 $0.0

Growth regular (sodium salicylate) 0 0 g $0.0 $0.0 $0.0

Herbicides (row middle only) 1 1 ha $70.0 $70.0 $70.0

Black plastic mulching 1 1 ha $1,353.6 $1,353.6 $1,353.6

Drip irrigation system 1 1 ha $279.1 $279.1 $279.1

Tomato seed 6 6 1000 $32.5 $195.0 $195.0

Irrigation (water) 1316 1316 ha m^3^ $0.1 $128.0 $128.0

Labor 1 1 ha $1,847.6 $1,847.6 $1,847.6

Machinery 1 1 ha $1,548.7 $1,548.7 $1,548.7

Total operating costs 1 1 ha $627.0 $6,281.0 $6,281.0

Cash overhead costs $316.2 $316.2

Non-cash overhead costs $496.5 $496.5

Total cost / hectare $7,093.6 $7,093.6

Total cost / ton $176.0 $163.1

Net return $523.1 $1,127.9

Benefit / cost 1.2 1.3

Economic productivity (kg / $) 5.7 6.1

**Energy equivalent Quantity /ha Unit Energy eq. Energy (MJ/ha)**

Energy input **2022 2023 (MJ/kg) 2022 2023**

Plastic mulch (kg / ha) 259 259 kg 158.0 40,922.0 40922.0

Chemicals (herbicides) 74 74 kg 101.2 7,488.8 7488.8

Nitrogen 202 202 kg 66.1 13,360.3 13360.3

Phosphorus 202 202 kg 12.4 2,512.9 2512.9

Potassium 202 202 kg 11.2 2,252.3 2252.3

Nanoliquid - NanoPro 0 0 mL 0.0 0.0 0.0

Growth regular (sodium salicylate) 0 0 g 0.01 0.0 0.0

Seeds 0.25 0.25 kg 1.0 0.3 0.3

Water 1316 1316 m^3^ 0.6 829.1 829.1

Labor 56 56 hr 2.0 109.8 109.8

Machinery 27.8 27.8 hr. 62.7 1,743.1 1743.1

Diesel 183 183 liter 56.3 10,304.7 10304.7

Total energy input 79,523.1 79523.1

Tomato energy output 40,300 43,500 kg 0.8 32,240.0 34800.0

Energy use efficiency (%) 40.5 43.8

Energy productivity (kg / MJ) 0.51 0.53

Net energy (MJ / ha) -47283.1 -44723.1

**Greenhouse gas (GHG) emissions Quantity /ha Unit GHG coefficient CO_2_ eq. /ha**

**2022 2023 (kg CO_2_-eq. unit^-1^) 2022 2023**

Plastic mulch use/disposal 259 259 kg 3.1 801.1 801.1

Plastic mulch manufacturing 259 259 kg 28 0.0 0.0

Chemicals 74 74 kg 2.47 182.8 182.8

Nitrogen 202 202 1.3 262.6 262.6

Phosphorus 202 202 0.2 40.4 40.4

Potassium 202 202 0.15 30.3 30.3

Nanoliquid - NanoPro 0 0 0 0 0.0 0.0

Growth regular (sodium salicylate) 50 50 g 2.23 0.0 0.0

Diesel 183 183 2.76 505.1 505.1

Machinery 1743.1 1743.1 0.071 123.8 123.8

Total GHG emission (kg CO_2_ / ha) 1946.0 1946.0

GHG intensity (kg CO_2_ / ton tomato 48.3 44.7

**S2 Table.** Economic, energy, and environmental assessment of processing tomato production with 40 kg of nano-NPK fertilization per ha in 2022 and 2023.

_____________________________________________________________________________________________

**Economics Quantity /ha Unit $/Unit Total ($/ha)**

**2022 2023 2022 2023**

Economic output 26.3 28.2 Ton $189.0 $4,970.7 $5,329.8

Economic input

Pre-plant fertilization (Triple 19 NPK) 105 105 kg $0.6 $59.0 $59.0

Drip fertilization (Triple 20 NPK) 100 100 kg $1.6 $160.0 $160.0

Nanoliquid - NanoPro 300 300 mL $0.0 $0.0 $0.0

Growth regular (sodium salicylate) 0 0 g $0.4 $0.0 $0.0

Herbicides (row middle only) 1 1 ha $70.0 $70.0 $70.0

Black plastic mulching 1 1 ha $1,353.6 $1,353.6 $1,353.6

Drip irrigation system 1 1 ha $279.1 $279.1 $279.1

Tomato seed 6 6 1000 $32.5 $195.0 $195.0

Irrigation (water) 1316 1316 ha m3 $0.1 $128.0 $128.0

Labor 1 1 ha $1,847.6 $1,847.6 $1,847.6

Machinery 1 1 ha $1,548.7 $1,548.7 $1,548.7

Total operating costs 1 1 ha $627.0 $5,641.0 $5,641.0

Cash overhead cost $316.2 $316.2

Non-cash overhead cost $496.5 $496.5

Total cost / hectare $6,453.6 $6,453.6

Total cost / ton $245.4 $228.9

Net return -$1,482.9 -$1,123.8

Benefit / cost 0.9 $1

Economic productivity (kg / $) 4.1 4.4

**Energy equivalent Quantity /ha Unit Energy eq. Energy (MJ/ha)**

Energy input **2022 2023 (MJ/kg) 2022 2023**

Plastic mulch (kg / ha) 259 259 kg 158.0 40,922.0 40922

Chemicals (herbicides) 74 74 kg 101.2 7,488.8 7,488.8

Nitrogen 68 68 kg 66.1 4,497.5 4,497.5

Phosphorus 68 68 kg 12.4 845.9 845.9

Potassium 68 68 kg 11.2 758.2 758.2

Nanoliquid - NanoPro 0 0 mL 0.0 0.0 0.0

Growth regular (sodium salicylate) 0 0 g 0.01 0.0 0.0

Seeds 0.25 0.25 kg 1.0 0.3 0.3

Water 1316 1316 m^3^ 0.6 829.1 829.1

Labor 56 56 hr 2.0 109.8 109.8

Machinery 27.8 27.8 hr. 62.7 1,743.1 1,743.1

Diesel 183 183 liter 56.3 10,304.7 10,304.7

Total energy input 67,499.3 67,499.3

Tomato energy output 26,300 28,200 kg 0.8 21,040.0 22,560.0

Energy use efficiency (%) 31.2 33.4

Energy productivity (kg / MJ) 0.62 0.6

Net energy (MJ / ha) -46459.3 -44939.32

**Greenhouse gas (GHG) emissions Quantity /ha Unit GHG coefficient CO_2_ eq. /ha**

**2022 2023 (kg CO_2_-eq. unit^-1^) 2022 2023**

Plastic mulch use/disposal 259 259 kg 3.1 801.1 801.1

Plastic mulch manufacturing 259 259 kg 28 0.0 0.0

Chemicals 74 74 kg 2.47 182.8 182.8

Nitrogen 68 68 1.3 88.4 88.4

Phosphorus 68 68 0.2 13.6 13.6

Potassium 68 68 0.15 10.2 10.2

Nanoliquid - NanoPro 0 0 0 0 0.0 0.0

Growth regular (sodium salicylate) 50 50 g 2.23 0.0 0.0

Diesel 183 183 2.76 505.1 505.1

Machinery 1743.1 1743.1 0.071 123.8 123.8

Total GHG emission (kg CO_2_ / ha) 1724.9 1,724.9

GHG intensity (kg CO_2_ / ton tomato) 65.6 61.2

**S3 Table.** Economic, energy, and environmental assessment of processing tomato production with 80 kg of nano-NPK fertilization per ha in 2022 and 2023.

________________________________________________________________________________________________________

**Economics Quantity /ha Unit $/Unit Total ($/ha)**

**2022 2023 2022 2023**

Economic output 39.5 46.5 Ton $189.0 $7,465.5 $8,788.5

Economic input

Pre-plant fertilization (Triple 19 NPK) 105 105 kg $0.6 $59.0 $59.0

Drip fertilization (Triple 20 NPK) 300 300 kg $1.6 $480.0 $480.0

Nanoliquid - NanoPro 300 300 mL $0.0 $0.0 $0.0

Growth regular (sodium salicylate) 0 0 g $0.4 $0.0 $0.0

Herbicides (row middle only) 1 1 ha $70.0 $70.0 $70.0

Black plastic mulching 1 1 ha $1,353.6 $1,353.6 $1,353.6

Drip irrigation system 1 1 ha $279.1 $279.1 $279.1

Tomato seed 6 6 1000 $32.5 $195.0 $195.0

Irrigation (water) 1316 1316 ha m^3^ $0.1 $128.0 $128.0

Labor 1 1 ha $1,847.6 $1,847.6 $1,847.6

Machinery 1 1 ha $1,548.7 $1,548.7 $1,548.7

Total operating costs 1 1 ha $627.0 $5,961.0 $5,961.0

Cash overhead cost $316.2 $316.2

Non-cash overhead cost $496.5 $496.5

Total cost / hectare $6,773.6 $6,773.6

Total cost / ton $171.5 $145.7

Net return $691.9 $2,014.9

Benefit / cost 1.3 1.5

Economic productivity (kg / $) 5.8 6.9

**Energy equivalent Quantity /ha Unit Energy eq. Energy (MJ/ha)**

Energy input **2022 2023 (MJ/kg) 2022 2023**

Plastic mulch (kg / ha) 259 259 kg 158.0 40,922.0 40,922.0

Chemicals (herbicides) 74 74 kg 101.2 7,488.8 7,488.8

Nitrogen 135 135 kg 66.1 8,928.9 8,928.9

Phosphorus 135 135 kg 12.4 1,679.4 1,679.4

Potassium 135 135 kg 11.2 1,505.3 1,505.3

Nanoliquid - NanoPro 0 0 mL 0.0 0.0 0.0

Growth regular (sodium salicylate) 0 0 g 0.01 0.0 0.0

Seeds 0.25 0.25 kg 1.0 0.3 0.3

Water 1316 1316 m^3^ 0.6 829.1 829.1

Labor 56 56 hr 2.0 109.8 109.8

Machinery 27.8 27.8 hr. 62.7 1,743.1 1,743.1

Diesel 183 183 liter 56.3 10,304.7 10,304.7

Total energy input 73,511.2 73,511.2

Tomato energy output 39,500 46,500 kg 0.8 31,600.0 37,200.0

Energy use efficiency (%) 43.0 50.6

Energy productivity (kg / MJ) 0.57 0.57

Net energy (MJ / ha) -41911.2 -36311.2

**Greenhouse gas (GHG) emissions Quantity /ha Unit GHG coefficient CO_2_ eq. /ha**

**2022 2023 (kg CO_2_-eq. unit^-1^) 2022 2023**

Plastic mulch use/disposal 259 259 kg 3.1 801.1 801.1

Plastic mulch manufacturing 259 259 kg 28 0.0 0.0

Chemicals 74 74 kg 2.47 182.8 182.8

Nitrogen 135 135 1.3 175.5 175.5

Phosphorus 135 135 0.2 27.0 27.0

Potassium 135 135 0.15 20.3 20.3

Nanoliquid - NanoPro 0 0 0 0 0.0 0.0

Growth regular (sodium salicylate) 50 50 g 2.23 0.0 0.0

Diesel 183 183 2.76 505.1 505.1

Machinery 1743.1 1743.1 0.071 123.8 123.8

Total GHG emission (kg CO_2_/ ha) 1835.5 1835.5

GHG intensity (kg CO_2_/ ton tomato) 46.5 39.5

**S4 Table.** Economic, energy, and environmental assessment of processing tomato production with 120 kg of nano-NPK fertilization per ha in 2022 and 2023.

________________________________________________________________________________________________________

**Economics Quantity /ha Unit $/Unit Total ($/ha)**

**2022 2023 2022 2023**

Economic output 42.5 48.7 Ton $189.0 $8,032.5 $9,204.3

Economic input

Pre-plant fertilization (Triple 19 NPK) 105 105 kg $0.6 $59.0 $59.0

Drip fertilization (Triple 20 NPK) 500 500 kg $1.6 $800.0 $800.0

Nanoliquid - NanoPro 300 300 mL $0.0 $8.1 $8.1

Growth regular (sodium salicylate) 0 0 g $0.01 $0.0 $0.0

Herbicides (row middle only) 1 1 ha $70.0 $70.0 $70.0

Black plastic mulching 1 1 ha $1,353.6 $1,353.6 $1,353.6

Drip irrigation system 1 1 ha $279.1 $279.1 $279.1

Tomato seed 6 6 1000 $32.5 $195.0 $195.0

Irrigation (water) 1507 1507 ha m^3^ $0.1 $146.6 $146.6

Labor 1 1 ha $1,847.6 $1,847.6 $1,847.6

Machinery 1 1 ha $1,548.7 $1,548.7 $1,548.7

Total operating costs $6,307.7 $6,307.7

Cash overhead cost $316.2 $316.2

Non-cash overhead cost $496.5 $496.5

Total cost / hectare $7,120.3 $7,120.3

Total cost / ton $167.5 $146.2

Net return $912.2 $2084.0

Benefit / cost 1.3 1.5

Economic productivity (kg / $) 6.0 6.8

**Energy equivalent Quantity /ha Unit Energy eq. Energy (MJ/ha)**

**2022 2023 (MJ/kg) 2022 2023**

Energy input

Plastic mulch (kg / ha) 259 259 kg 158.0 40,922.0 40,922.0

Chemicals (herbicides) 74 74 kg 101.2 7,488.8 7,488.8

Nitrogen 202 202 kg 66.1 13,360.3 13,360.3

Phosphorus 202 202 kg 12.4 2,512.9 2,512.9

Potassium 202 202 kg 11.2 2,252.3 2,252.3

Nanoliquid - NanoPro 300 300 mL 0.0 0.0 0.0

Growth regular (sodium salicylate) 0 0 g 0.01 0.0 0.0

Seeds 0.25 0.25 kg 1.0 0.3 0.3

Water 1316 1316 m^3^ 0.6 829.1 829.1

Labor 56 56 hr 2.0 109.8 109.8

Machinery 27.8 27.8 hr. 62.7 1,743.1 1,743.1

Diesel 183 183 liter 56.3 10,304.7 10,304.7

Total energy input 79,523.1 79,523.1

Tomato energy output 42,500 48,700 kg 0.8 34,000.0 38,960.0

Energy use efficiency (%) 42.8 49.0

Energy productivity (kg / MJ) 0.53 0.53

Net energy (MJ / ha) -45523.1 -40563.1

**Greenhouse gas (GHG) emissions Quantity /ha Unit GHG coefficient CO_2_ eq. /ha**

**2022 2023 (kg CO_2_-eq. unit^-1^) 2022 2023**

Plastic mulch use/disposal 259 259 kg 3.1 801.1 801.1

Plastic mulch manufacturing 259 259 kg 28 0.0 0.0

Chemicals 74 74 kg 2.47 182.8 182.8

Nitrogen 202 202 1.3 262.6 262.6

Phosphorus 202 202 0.2 40.4 40.4

Potassium 202 202 0.15 30.3 30.3

Nanoliquid - NanoPro 0 0 0 0 0.0 0.0

Growth regular (sodium salicylate) 50 50 g 2.23 0.1 0.1

Diesel 183 183 2.76 505.1 505.1

Machinery 1743.1 1743.1 0.071 123.8 123.8

Total GHG emission (kg CO_2_ / ha) 1946.1 1946.1

GHG intensity (kg CO_2_ / ton tomato 45.8 40.0

**S5 Table.** Economic, energy, and environmental assessment of processing tomato production with 120 kg NPK fertilization per ha and 0.3 percent salicylic acid application in 2022 and 2023.

________________________________________________________________________________________________________

**Economics Quantity /ha Unit $/Unit Total ($/ha)**

**2022 2023 2022 2023**

Economic out 45.6 45.8 Ton $189.0 $8,618.4 $8,656.2

Economic input

Pre-plant fertilization (Triple 19 NPK) 105 105 kg $0.6 $59.0 $59.0

Drip fertilization (Triple 20 NPK) 500 500 kg $1.6 $800.0 $800.0

Nanoliquid - NanoPro 300 300 mL $0.0 $0.0 $0.0

Growth regular (sodium salicylate) 50 50 g $0.01 $0.4 $0.4

Herbicides (row middle only) 1 1 ha $70.0 $70.0 $70.0

Black plastic mulching 1 1 ha $1,353.6 $1,353.6 $1,353.6

Drip irrigation system 1 1 ha $279.1 $279.1 $279.1

Tomato seed 6 6 1000 $32.5 $195.0 $195.0

Irrigation (water) 1143 1143 ha m^3^ $0.1 $111.2 $111.2

Labor 1 1 ha $1,847.6 $1,847.6 $1,847.6

Machinery 1 1 ha $1,548.7 $1,548.7 $1,548.7

Total operating cost $6,264.5 $6,264.5

Cash overhead cost $316.2 $316.2

Non-cash overhead cost $496.5 $496.5

Total cost / hectare $7,077.2 $7,077.2

Total cost / ton $155.2 $154.5

Net return $1,541.2 $1,579.0

Benefit / cost 1.4 1.4

Economic productivity (kg / $) 6.4 6.5

**Energy equivalent Quantity /ha Unit Energy eq. Energy (MJ/ha)**

**2022 2023 (MJ/kg) 2022 2023**

Energy input

Plastic mulch (kg / ha) 259 259 kg 158.0 40,922.0 40,922.0

Chemicals (herbicides) 74 74 kg 101.2 7,488.8 7,488.8

Nitrogen 202 202 kg 66.1 13,360.3 13,360.3

Phosphorus 202 202 kg 12.4 2,512.9 2,512.9

Potassium 202 202 kg 11.2 2,252.3 2,252.3

Nanoliquid - NanoPro 300 300 mL 0.0 0.0 0.0

Growth regular (sodium salicylate) 50 50 g 0.01 0.4 0.4

Seeds 0.25 0.25 kg 1.0 0.3 0.3

Water 1316 1316 m^3^ 0.6 829.1 829.1

Labor 56 56 hr 2.0 109.8 109.8

Machinery 27.8 27.8 hr. 62.7 1,743.1 1,743.1

Diesel 183 183 liter 56.3 10,304.7 10,304.7

Total energy input 79,523.5 79,523.5

Tomato energy output 45,600 45,800 kg 0.8 36,480.0 36,640.0

Energy use efficiency (%) 45.9 46.1

Energy productivity (kg / MJ) 0.53 0.53

Net energy (MJ / ha) -43043.5 -42883.5

**Greenhouse gas (GHG) emissions Quantity /ha Unit GHG coefficient CO_2_ eq. /ha**

**2022 2023 (kg CO_2_-eq. unit^-1^) 2022 2023**

Plastic mulch use/disposal 259 259 kg 3.1 801.1 801.1

Plastic mulch manufacturing 259 259 kg 28 0.0 0.0

Chemicals 74 74 kg 2.47 182.8 182.8

Nitrogen 202 202 1.3 262.6 262.6

Phosphorus 202 202 0.2 40.4 40.4

Potassium 202 202 0.15 30.3 30.3

Nanoliquid - NanoPro 0 0 0 0 0.0 0.0

Growth regular (sodium salicylate) 0 0 g 2.23 0.0 0.0

Diesel 183 183 2.76 505.1 505.1

Machinery 1743.1 1743.1 0.071 123.8 123.8

Total GHG emission (kg CO_2_ / ha) 1946.0 1946.0

GHG intensity (kg CO_2_ / ton tomato) 42.7 42.5

**S6 Table.** Economic, energy, and environmental assessment of processing tomato production with 40 kg nano-NPK fertilization per ha and 0.3 percent salicylic acid application in 2022 and 2023.

_____________________________________________________________________________________________

**Economics Quantity /ha Unit $/Unit Total ($/ha)**

**2022 2023 2022 2023**

Economic output 29 33.8 Ton $189 $5,481.0 $6,388.2

Economic input

Pre-plant fertilization (Triple 19 NPK) 105 105 kg $0.6 $59.0 $59.0

Drip fertilization (Triple 20 NPK) 100 100 kg $1.6 $160.0 $160.0

Nanoliquid - NanoPro 300 300 mL $0.0 $0 $0

Growth regular (sodium salicylate) 50 50 g $0.01 $0.4 $0.4

Herbicides (row middle only) 1 1 ha $70.0 $70.0 $70.0

Black plastic mulching 1 1 ha $1353.6 $1,353.6 $1,353.6

Drip irrigation system 1 1 ha $279.1 $279.1 $279.1

Tomato seed 6 6 1000 $32.5 $195.0 $195.0

Irrigation (water) 1143 1143 ha m^3^ $0.1 $111.2 $111.2

Labor 1 1 ha $1847.6 $1,847.6 $1,847.6

Machinery 1 1 ha $1548.7 $1,548.7 $1,548.7

Total operating cost $5,624.5 $5,624.5

Cash overhead cost $316.2 $316.2

Non-cash overhead cost $496.5 $496.5

Total cost / hectare $6,437.2 $6,437.2

Total cost / ton $222.0 $190.4

Net return -$956.2 -$49.0

Benefit / cost 1.0 1.1

Economic productivity (kg / $) 4.5 5.3

**Energy equivalent Quantity/ha Unit Energy eq. Energy (MJ/ha)**

**2022 2023 (MJ/kg) 2022 2023**

Energy input

Plastic mulch (kg / ha) 259 259 kg 158.0 40,922.0 40,922.0

Chemicals (herbicides) 74 74 kg 101.2 7,488.8 7,488.8

Nitrogen 68 68 kg 66.1 4,497.5 4,497.5

Phosphorus 68 68 kg 12.4 845.9 845.9

Potassium 68 68 kg 11.2 758.2 758.2

Nanoliquid - NanoPro 300 300 mL 0.0 0.0 0.0

Growth regular (sodium salicylate) 50 50 g 0.01 0.4 0.4

Seeds 0.25 0.25 kg 1.0 0.3 0.3

Water 1316 1316 m^3^ 0.6 829.1 829.1

Labor 56 56 hr 2.0 109.8 109.8

Machinery 27.8 27.8 hr. 62.7 1,743.1 1,743.1

Diesel 183 183 liter 56.3 10,304.7 10,304.7

Total energy input 67,499.7 67,499.7

Tomato energy output 29,000 33,800 kg 0.8 23,200.0 27,040.0

Energy use efficiency (%) 34.4 40.1

Energy productivity (kg / MJ) 0.6 0.6

Net energy (MJ / ha) -44299.7 -40459.7

**Greenhouse gas (GHG) emissions Quantity /ha Unit GHG coefficient CO_2_ eq. /ha**

**2022 2023 (kg CO_2_-eq. unit^-1^) 2022 2023**

Plastic mulch use/disposal 259 259 kg 3.1 801.1 801.1

Plastic mulch manufacturing 259 259 kg 28 0.0 0.0

Chemicals 74 74 kg 2.47 182.8 182.8

Nitrogen 68 68 1.3 88.4 88.4

Phosphorus 68 68 0.2 13.6 13.6

Potassium 68 68 0.15 10.2 10.2

Nanoliquid - NanoPro 0 0 0 0 0.0 0.0

Growth regular (sodium salicylate) 0 0 g 2.23 0.0 0.0

Diesel 183 183 2.76 505.1 505.1

Machinery 1743.1 1743.1 0.071 123.8 123.8

Total GHG emission (kg CO_2_ / ha) 1724.9 1724.9

GHG intensity (kg CO_2_ / ton tomato) 59.5 51.0

**S7 Table.** Economic, energy, and environmental assessment of processing tomato production with 80 kg nano-NPK fertilization per ha and 0.3 percent salicylic acid application in 2022 and 2023.

_____________________________________________________________________________________________

**Economics Quantity/ha Unit $/Unit Total ($/ha)**

**2022 2023 2022 2023**

Economic output 45 47.8 Ton $189 $8,505 $9,034.2

Economic input

Pre-plant fertilization (Triple 19 NPK) 105 105 kg $0.6 $59.0 $59.0

Drip fertilization (Triple 20 NPK) 300 300 kg $1.6 $480.0 $480

Nanoliquid - NanoPro 300 300 mL $0.0 $0 $0

Growth regular (sodium salicylate) 50 50 g $0.01 $0.4 $0.4

Herbicides (row middle only) 1 1 ha $70.0 $70.0 $70.0

Black plastic mulching 1 1 ha $1,353.6 $1,353.6 $1,353.6

Drip irrigation system 1 1 ha $279.1 $279.1 $279.1

Tomato seed 6 6 1000 $32.5 $195.0 $195.0

Irrigation (water) 1,143 1,143 ha m^3^ $0.1 $111.2 $111.2

Labor 1 1 ha $1,847.6 $1,847.6 $1,847.8

Machinery 1 1 ha $1,548.7 $1,548.7 $1,548.7

Total operating cost $5,944.5 $5,944.5

Cash overhead cost $316.2 $316.2

Non-cash overhead cost $496.5 $496.5

Total cost / hectare $6,757.2 $6,757.2

Total cost / ton $150.2 $141.4

Net return $1,747.8 $2,277

Benefit / cost 1.4 1.5

Economic productivity (kg / $) 6.7 7.1

**Energy equivalent Quantity /ha Unit Energy eq. Energy (MJ/ha)**

**2022 2023 (MJ/kg) 2022 2023**

Energy input

Plastic mulch (kg / ha) 259 259 kg 158.0 40,922.0 40,922.0

Chemicals (herbicides) 74 74 kg 101.2 7,488.8 7,488.8

Nitrogen 135 135 kg 66.1 8,928.9 8,928.9

Phosphorus 135 135 kg 12.4 1,679.4 1,679.4

Potassium 135 135 kg 11.2 1,505.3 1,505.3

Nanoliquid - NanoPro 300 300 mL 0.0 0.0 0.0

Growth regular (sodium salicylate) 50 50 g 0.01 0.4 0.4

Seeds 0.25 0.25 kg 1.0 0.3 0.3

Water 1316 1316 m3 0.6 829.1 829.1

Labor 56 56 hr 2.0 109.8 109.8

Machinery 27.8 27.8 hr. 62.7 1,743.1 1,743.1

Diesel 183 183 liter 56.3 10,304.7 10,304.7

Total energy input 73,511.6 73,511.6

Tomato energy output 45,000 47,800 kg 0.8 36,000.0 38,240.0

Energy use efficiency (%) 49.0 52.0

Energy productivity (kg / MJ) 0.57 0.57

Net energy (MJ / ha) -37511.6 -35271.6

**Greenhouse gas (GHG) emissions Quantity /ha Unit GHG coefficient CO_2_ eq. /ha**

**2022 2023 (kg CO_2_-eq. unit^-1^) 2022 2023**

Plastic mulch use/disposal 259 259 kg 3.1 801.1 801.1

Plastic mulch manufacturing 259 259 kg 28 0.0 0.0

Chemicals 74 74 kg 2.47 182.8 182.8

Nitrogen 135 135 1.3 175.5 175.5

Phosphorus 135 135 0.2 27.0 27.0

Potassium 135 135 0.15 20.3 20.3

Nanoliquid - NanoPro 0 0 0 0 0.0 0.0

Growth regular (sodium salicylate) 0 0 g 2.23 0.0 0.0

Diesel 183 183 2.76 505.1 505.1

Machinery 1,743.1 1,743.1 0.071 123.8 123.8

Total GHG emission (kg CO_2_ / ha) 1,835.5 1,835.5

GHG intensity (kg CO_2_ / ton tomato) 40.8 38.4

**S8 Table.** Economic, energy, and environmental assessment of processing tomato production with 120 kg nano-NPK fertilization per ha and 0.3 percent salicylic acid application in 2022 and 2023.

________________________________________________________________________________________________________

**Economics Quantity/ha Unit $/Unit Total ($/ha)**

**2022 2023 2022 2023**

Economic output 48 53.5 Ton $189 $9,072.0 10111.5

Economic input

Pre-plant fertilization (Triple 19 NPK) 105 105 kg $0.6 $59.0 $59.0

Drip fertilization (Triple 20 NPK) 500 500 kg $1.6 $800.0 $80.0

Nanoliquid - NanoPro 300 300 mL $2.7 $8.0 $8.0

Growth regular (sodium salicylate) 50 50 g $0.03 $1.6 $1.6

Herbicides (row middle only) 1 1 ha $70.0 $70.0 $70.0

Black plastic mulching 1 1 ha $1353.6 $1,353.6 $1,353.6

Drip irrigation system 1 1 ha $279.1 $279.1 $279.1

Tomato seed 6 6 1000 $32.5 $195.0 $195.0

Irrigation (water) 1,424 1,424 ha m^3^ $0.1 $138.6 $138.6

Labor 1 1 ha $1847.6 $1,847.6 $1,847.6

Machinery 1 1 ha $1548.7 $1,548.7 $1,548.7

Total operating cost $6,301.1 $6,301.1

Cash overhead cost $316.2 $3,16.2

Non-cash overhead cost $496.5 $4,96.5

Total cost / hectare $7,113.7 $7,113.7

Total cost / ton $148.2 $133.0

Net return $1,958.3 $2,997.8

Benefit / Cost 1.4 1.6

Economic productivity (kg / $) 6.7 7.5

**Energy equivalent Quantity /ha Unit Energy eq. Energy (MJ/ha)**

**2022 2023 (MJ/kg) 2022 2023**

Energy input

Plastic mulch (kg/ha) 259 259 kg 158.0 40,922.0 40,922.0

Chemicals (herbicides) 74 74 kg 101.2 7,488.8 7,488.8

Nitrogen 202 202 kg 66.1 13,360.3 13,360.3

Phosphorus 202 202 kg 12.4 2,512.9 2,512.9

Potassium 202 202 kg 11.2 2,252.3 2,252.3

Nanoliquid - NanoPro 300 300 mL 0.0 0.0 0

Growth regular (sodium salicylate) 50 50 g 0.01 0.4 0.4

Seeds 0.25 0.25 kg 1.0 0.3 0.3

Water 1,316 1,316 m^3^ 0.6 829.1 829.1

Labor 56 56 hr 2.0 109.8 109.8

Machinery 27.8 27.8 hr. 62.7 1,743.1 1,743.1

Diesel 183 183 liter 56.3 10,304.7 1,0304.7

Total energy input 79,523.5 79,523.5

Tomato energy output 48,000 53,500 kg 0.8 38,400.0 42,800.0

Energy use efficiency (%) 48.3 53.8

Energy productivity (kg / MJ) 0.53 0.53

Net energy (MJ / ha) -41,123.5 -36,723.5

**Greenhouse gas (GHG) emissions Quantity /ha Unit GHG coefficient CO_2_ eq. /ha**

**2022 2023 (kg CO_2_-eq. unit^-1^) 2022 2023**

Plastic mulch use/disposal 259 259 kg 3.1 801.1 801.1

Plastic mulch manufacturing 259 259 kg 28 0.0 0

Chemicals 74 74 kg 2.47 182.8 182.8

Nitrogen 202 202 1.3 262.6 262.6

Phosphorus 202 202 0.2 40.4 40.4

Potassium 202 202 0.15 30.3 30.3

Nanoliquid - NanoPro 0 0 0 0 0.0 0

Growth regular (sodium salicylate) 50 50 g 2.23 0.1 0.1

Diesel 183 183 2.76 505.1 505.1

Machinery 1743.1 1743.1 0.071 123.8 123.8

Total GHG emission (kg CO_2_ / ha) 1,946.2 1,946.2

GHG intensity (kg CO_2_ / ton tomato) 40.5 36.4
