# Supplementary material for: Improving tomato yield and production economics with Nano-NPK fertilization and salicylic acid chemigation
Source: PLoS One. 2026 May 4;21(5):e0348207. doi: 10.1371/journal.pone.0348207 (PMC13138637; doi:10.1371/journal.pone.0348207)
Supplement: Attachment — Submitted filename: PONE-D-25-44279.pdf [file pone.0348207.s004.pdf]

PLOS ONE

Improving Tomato Yield and Production Economics with Nano-NPK Fertilization and Salicylic Acid Chemigation

--Manuscript Draft--

**Manuscript Number:**	PONE-D-25-44279
**Article Type:**	Research Article
**Full Title:**	Improving Tomato Yield and Production Economics with Nano-NPK Fertilization and Salicylic Acid Chemigation
**Short Title:**	Tomato Yield and Production Economics with Nano-NPK Fertilization and Salicylic Acid
**Corresponding Author:**	Khandakar Rafiq Islam, Ph.D.<br>The Ohio State University Extension<br>Piketon, OH UNITED STATES OF AMERICA
**Keywords:**	Marketable yield, Fertilizer-use efficiency, Economic productivity, Energy-use efficiency, Greenhouse gas intensity.
**Abstract:**	Nanotechnology-based fertilization combined with salicylic acid (SA) offers a promising, sustainable strategy to boost crop productivity, resource-use efficiency, and environmental performance in specialty crops. This two-year field study (2022–2023) evaluated the interactive effects of nano humic acid–silicic acid-mixed NPK fertilizers (nano-NPK) and SA chemigation on processing tomato (Solanum lycopersicum L. cv. BHN 685) production under drip-irrigated raised bed plasticulture. A 3 × 2 factorial experiment in complete randomized design with four replications was used to assess three nano-NPK rates (40, 80, and 120 kg ha^{-1}) with and without 0.3% SA, compared to a conventional NPK control (120 kg ha^{-1}). Nano-NPK at 80 and 120 kg ha^{-1} significantly increased total and marketable yields by up to 10% and 17%, primarily by reducing cull fruits by 11–23%. SA alone enhanced yields by 10–15%. The combined 80 kg ha^{-1} nano-NPK and SA treatment matched or exceeded the 120 kg NPK or 120 kg nano-NPK ha-1, indicating potential input reduction without yield loss. Water-use efficiency (WUE) improved by 13–34% with increasing nano-NPK, and by an additional 11% with SA. Although fertilizer-use efficiency (FUE) declined at higher NPK or nano-NPK rates, it remained up to 61% higher than the control and improved by 23% with SA. Regression analysis showed a non-linear WUE increase and FUE decline with increasing nano-NPK rates, with the 80 kg ha^{-1} + SA treatment offering the best balance. Economic analysis showed slight cost increases with nano-NPK and SA, but substantial gains in economic output, benefit–cost ratio, and net income. The highest net return ($2,478 ha^{-1}) and benefit–cost ratio (1.5) were recorded with 80–120 kg ha^{-1} nano-NPK + SA. While all treatments had net negative energy use, nano-NPK + SA reduced it by up to 9,611 MJ ha^{-1} and improved energy efficiency by 8.9%. Greenhouse gas intensity was lower with nano-NPK, confirming environmental benefits. Overall, applying 80 kg nano-NPK ha^{-1} with 0.3% SA is an effective strategy to improve tomato yield, efficiency, and sustainability.
**Order of Authors:**	Khandakar Rafiq Islam, Ph.D.
	Zhenhao Guan, MS
	Arifur Rahman, Ph.D.
	Brad Bergefurd, MS
**Additional Information:**	
**Question**	**Response**
**Financial Disclosure**<br><br>Enter a financial disclosure statement that describes the sources of funding for the work included in this submission. Review	Yes

Powered by Editorial Manager® and ProduXion Manager® from Aries Systems Corporation

the submission guidelines for detailed requirements. View published research articles from *PLOS ONE* for specific examples.<br><br>This statement is required for submission and **will appear in the published article** if the submission is accepted. Please make sure it is accurate.<br><br>**Funded studies**<br>Enter a statement with the following details:<br>• Initials of the authors who received each award<br>• Grant numbers awarded to each author<br>• The full name of each funder<br>• URL of each funder website<br>• Did the sponsors or funders play any role in the study design, data collection and analysis, decision to publish, or preparation of the manuscript?<br><br>Did you receive funding for this work?	
Please add funding details.<br>as follow-up to "**Financial Disclosure**<br><br>Enter a financial disclosure statement that describes the sources of funding for the work included in this submission. Review the submission guidelines for detailed requirements. View published research articles from *PLOS ONE* for specific examples.<br><br>This statement is required for submission and **will appear in the published article** if the submission is accepted. Please make sure it is accurate.	The financial support provided by the Ohio Department of Agriculture via USDA Specialty Crops Block grant (2021-2024)

**Funded studies**<br>Enter a statement with the following details:<br>• Initials of the authors who received each award<br>• Grant numbers awarded to each author<br>• The full name of each funder<br>• URL of each funder website<br>• Did the sponsors or funders play any role in the study design, data collection and analysis, decision to publish, or preparation of the manuscript?<br><br>Did you receive funding for this work?"	
Please select the country of your main research funder (please select carefully as in some cases this is used in fee calculation).<br>as follow-up to "**Financial Disclosure**<br><br>Enter a financial disclosure statement that describes the sources of funding for the work included in this submission. Review the submission guidelines for detailed requirements. View published research articles from *PLOS ONE* for specific examples.<br><br>This statement is required for submission and **will appear in the published article** if the submission is accepted. Please make sure it is accurate.<br><br>**Funded studies**<br>Enter a statement with the following details:<br>• Initials of the authors who received each award<br>• Grant numbers awarded to each author<br>• The full name of each funder<br>• URL of each funder website<br>• Did the sponsors or funders play any role in the study design, data collection and analysis, decision to publish, or preparation of the manuscript?<br><br>Did you receive funding for this work?"	UNITED STATES - US
**Competing Interests**	N/A

Use the instructions below to enter a competing interest statement for this submission. On behalf of all authors, disclose any competing interests that could be perceived to bias this work—acknowledging all financial support and any other relevant financial or non-financial competing interests.<br><br>This statement is required for submission and **will appear in the published article** if the submission is accepted. Please make sure it is accurate and that any funding sources listed in your Funding Information later in the submission form are also declared in your Financial Disclosure statement.<br><br>View published research articles from *PLOS ONE* for specific examples.<br><br>**NO authors have competing interests**<br><br>Enter: *The authors have declared that no competing interests exist.*<br><br>**Authors with competing interests**<br><br>Enter competing interest details beginning with this statement:<br><br>*I have read the journal's policy and the authors of this manuscript have the following competing interests: [insert competing interests here]*<br><br>* typeset	
**Ethics Statement**<br><br>Enter an ethics statement for this submission. This statement is required if the study involved:<br><br>• Human participants<br>• Human specimens or tissue<br>• Vertebrate animals or cephalopods<br>• Vertebrate embryos or tissues	N/A

• Field research<br><br>Write "N/A" if the submission does not require an ethics statement.<br><br>General guidance is provided below. Consult the submission guidelines for detailed instructions. **Make sure that all information entered here is included in the Methods section of the manuscript.**	

Powered by Editorial Manager® and ProduXion Manager® from Aries Systems Corporation

**Format for specific study types**<br><br>**Human Subject Research (involving human participants and/or tissue)**<br>• Give the name of the institutional review board or ethics committee that approved the study<br>• Include the approval number and/or a statement indicating approval of this research<br>• Indicate the form of consent obtained (written/oral) or the reason that consent was not obtained (e.g. the data were analyzed anonymously)<br><br>**Animal Research (involving vertebrate animals, embryos or tissues)**<br>• Provide the name of the Institutional Animal Care and Use Committee (IACUC) or other relevant ethics board that reviewed the study protocol, and indicate whether they approved this research or granted a formal waiver of ethical approval<br>• Include an approval number if one was obtained<br>• If the study involved *non-human primates*, add *additional details* about animal welfare and steps taken to ameliorate suffering<br>• If anesthesia, euthanasia, or any kind of animal sacrifice is part of the study, include briefly which substances and/or methods were applied<br><br>**Field Research**<br><br>Include the following details if this study involves the collection of plant, animal, or other materials from a natural setting:<br>• Field permit number<br>• Name of the institution or relevant body that granted permission	
**Data Availability**<br><br>Authors are required to make all data underlying the findings described fully available, without restriction, and from the time of publication. PLOS allows rare exceptions to address legal and ethical concerns. See the PLOS Data Policy and FAQ for detailed information.	Yes - all data are fully available without restriction

A Data Availability Statement describing where the data can be found is required at submission. Your answers to this question constitute the Data Availability Statement and **will be published in the article**, if accepted.<br><br>**Important:** Stating 'data available on request from the author' is not sufficient. If your data are only available upon request, select 'No' for the first question and explain your exceptional situation in the text box.<br><br>Do the authors confirm that all data underlying the findings described in their manuscript are fully available without restriction?	
**Describe where the data may be found in full sentences. If you are copying our sample text, replace any instances of XXX with the appropriate details.**<br><br>• If the data are **held or will be held in a public repository**, include URLs, accession numbers or DOIs. If this information will only be available after acceptance, indicate this by ticking the box below. For example: *All XXX files are available from the XXX database (accession number(s) XXX, XXX.*).<br>• If the data are all contained **within the manuscript and/or Supporting Information files**, enter the following: *All relevant data are within the manuscript and its Supporting Information files.*<br>• If neither of these applies but you are able to provide **details of access elsewhere**, with or without limitations, please do so. For example:<br><br>*Data cannot be shared publicly because of [XXX]. Data are available from the XXX Institutional Data Access / Ethics Committee (contact via XXX) for researchers who meet the criteria for access to confidential data.*<br><br>*The data underlying the results presented in the study are available from (include the name of the third party*	All relevant data are within the manuscript and its Supporting Information files.

and contact information or URL).<br>• This text is appropriate if the data are owned by a third party and authors do not have permission to share the data.<br><br>* typeset	
Additional data availability information:	

Manuscript

Click here to access/download;Manuscript;Nano NPK and SA paper 2025.docx

Improving Tomato Yield and Production Economics with Nano-NPK Fertilization and Salicylic Acid Chemigation

Islam Khandakar
Soil, Water, and Bioenergy Resources, The Ohio State University South Centers, Piketon, OH, USA. Orcid # 0000-0001-9332-5493.

Guan Zhenhao
Soil, Water, and Bioenergy Resources, The Ohio State University South Centers, Piketon, OH, USA. Orcid # 0009-0002-8818-2533.

Rahman Arifur
Soil, Water, and Bioenergy Resources, The Ohio State University South Centers, Piketon, OH, USA. Orcid # 0000-0002-5215-3506.

Bergefurd Bradford
Brandt Discovery & Innovations, LLC, Wilmington, OH, USA.
Orcid # 0000-0003-0825-8244.

Corresponding author:
Rafiq Islam,
The Ohio State University South Centers
Piketon, OH, USA

Abstract

Nanotechnology-based fertilization combined with salicylic acid (SA) offers a promising, sustainable strategy to boost crop productivity, resource-use efficiency, and environmental performance in specialty crops. This two-year field study (2022–2023) evaluated the interactive effects of nano humic acid–silicic acid-mixed NPK fertilizers (nano-NPK) and SA chemigation on processing tomato (*Solanum lycopersicum* L. cv. BHN 685) production under drip-irrigated raised bed plasticulture. A 3 × 2 factorial experiment in complete randomized design with four replications was used to assess three nano-NPK rates (40, 80, and 120 kg ha^{-1}) with and without 0.3% SA, compared to a conventional NPK control (120 kg ha^{-1}). Nano-NPK at 80 and 120 kg ha^{-1} significantly increased total and marketable yields by up to 10% and 17%, primarily by reducing cull fruits by 11–23%. SA alone enhanced yields by 10–15%. The combined 80 kg ha^{-1} nano-NPK and SA treatment matched or exceeded the 120 kg NPK or 120 kg nano-NPK ha^{-1}, indicating potential input reduction without yield loss. Water-use efficiency (WUE) improved by 13–34% with increasing nano-NPK, and by an additional 11% with SA. Although fertilizer-use efficiency (FUE) declined at higher NPK or nano-NPK rates, it remained up to 61% higher than the control and improved by 23% with SA. Regression analysis showed a non-linear WUE increase and FUE decline with increasing nano-NPK rates, with the 80 kg ha^{-1} + SA treatment offering the best balance. Economic analysis showed slight cost increases with nano-NPK and SA, but substantial gains in economic output, benefit–cost ratio, and net income. The highest net return ($2,478 ha^{-1}) and benefit–cost ratio (1.5) were recorded with 80–120 kg ha^{-1} nano-NPK + SA. While all treatments had net negative energy use, nano-NPK + SA reduced it by up to 9,611 MJ ha^{-1} and improved energy efficiency by 8.9%. Greenhouse gas intensity was lower with nano-NPK, confirming environmental benefits. Overall, applying 80 kg nano-NPK ha^{-1} with 0.3% SA is an effective strategy to improve tomato yield, efficiency, and sustainability.

Key words: Marketable yield, Fertilizer-use efficiency, Economic productivity, Energy-use efficiency, Greenhouse gas intensity.

Introduction

Tomato is the third most economically important crop globally after grasses and legumes, and it is the most valuable and widely cultivated specialty crop, with an annual production of approximately 182.3 million tons on about 4.85 million hectares of land [1]. Renowned for its unique culinary applications, tomato is consumed both fresh and processed products, serving as an important component of a nutritious diet and contributing to public health [2-3]. Processed tomato products—including purées, juices, ketchup, canned, and dried tomatoes—are economically valuable and continue to be used in response to increasing global demand. To meet this demand, tomatoes are cultivated across a range of soils, environments, and management practices, from open fields to controlled conditions such as greenhouses and high tunnels.

As with other specialty crops, achieving higher tomato yields largely depends on the use of inputs such as chemical fertilizers, organic and inorganic amendments, organo-chemical mixtures, and natural products [4]. However, commercial fertilizers—typically applied as nitrogen (N), phosphorus (P), and potassium (K)—exhibit low to moderate nutrient use efficiency (NUE), with efficiency rates of approximately 30–35% for N, 18–20% for P, and 35–40% for K [4]. These inefficiencies necessitate excessive fertilization, particularly of reactive chemical forms, to maintain productivity. This practice contributes to economic and environmental concerns, including nutrient leaching, water pollution with harmful algal blooms, greenhouse gas emissions, and the accumulation of heavy metals in soils and crops [5].

Excessive reliance on chemical fertilization not only affects agroecosystem services but also increases production costs related to energy, labor, materials, nutrients, and irrigation—ultimately reducing farm sustainability. Moreover, tomato production is increasingly challenged by abiotic and biotic stresses driven by global climate change, as well as by the growing scarcity of freshwater resources [5-6]. Improper irrigation—either insufficient or excessive—can hinder tomato growth, nutrient uptake, and overall yield. In water-scarce regions with low soil fertility, optimizing irrigation and fertilization is essential for sustainable tomato production. These challenges underscore the urgent need for innovative strategies to enhance tomato productivity while improving water-use efficiency (WUE) and NUE [6-7].

Nano-technology offers a promising strategy for precision and improved nutrient management, aiming to enhance crop productivity through controlled and efficient nutrient delivery [8-10]. Nano-materials—ranging in size from 1 to 100 nm—act as concentrated sources

or carriers of essential or beneficial nutrients. Their high surface area-to-volume ratio and unique physicochemical properties enable effective nutrient absorption and targeted delivery to plants [11-12]. Nanopores in plant roots facilitate the efficient uptake and translocation of nano-enabled fertilizers, thereby improving NUE.

Previous studies have demonstrated that nano-enabled fertilizers, whether applied to plants independently or in combination with chemical fertilizers, significantly improve the nutrient uptake by plants to sustain yield and quality compared to the use of fertilizers alone [3, 10, 13]. For example, the integration of FeO_x or SiO_2 nanoparticles with NPK fertilizers has been shown to increase tomato yield, enhance phytochemical quality, and improve WUE and NUE, especially when compared to conventional NPK formulations alone [3, 14-16]. These improvements are attributed to the nanoparticles' enhanced absorption, faster reactivity, greater utilization efficiency, and reduced nutrient losses—ultimately increasing yield while minimizing environmental footprints [17-18].

Alongside nano-fertilization, salicylic acid (SA)—a naturally occurring plant hormone and key stress-signaling molecule—has emerged as a promising tool to alleviate both abiotic and biotic stressors in plants [19-23]. When applied in dilute concentrations, SA enhances plant resilience to stress conditions such as drought, salinity, heavy metal toxicity, and pathogenic infections [22]. It does so by modulating a range of physiological and biochemical pathways, including stomatal behavior, transpiration rate, nutrient and water uptake efficiency [19, 24]. In addition, SA is known to stimulate the production of secondary metabolites like antioxidants and phytochemicals, which may contribute to improved plant vigor, fruit development, yield, and nutritional composition in vegetable crops including tomatoes [3, 25-26].

To advance sustainable tomato production, it is critical to adopt an integrated evaluation framework encompassing economic viability, energy efficiency, and environmental impact [27-29]. Economic analysis plays a fundamental role in promoting the adoption of sustainable practices in managed tomato production systems [30]. Likewise, energy efficiency remains a crucial metric in assessing agricultural productivity and sustainability [31]. Enhancing energy efficiency not only conserves valuable resources but also improves farm profitability and reduces environmental burdens [32]. Among environmental concerns, greenhouse gas (GHG) emissions from inputs and agricultural systems are of global significance. Reducing GHG emissions is a strategic approach to achieving climate goals, particularly the stabilization of carbon dioxide emissions before 2030 [33].

Although several studies have addressed the economic, energy, and environmental dimensions of agricultural systems—mainly focusing on cereal crops, as well as horticultural, and other crops [30, 34-35]—the combined impact of nanomaterial-enhanced NPK fertilization (nano-NPK) and SA chemigation on processing tomato production has not been explored. Investigating the potential synergistic benefits of this integrated approach on yield, profitability, energy use, and environmental sustainability represents a valuable and timely research opportunity.

This study aims to address that gap by evaluating the integrated application of nano-NPK fertilizers and SA chemigation in processing tomato production in low fertility soils. Specifically, the objectives were to: (1) assess the effects of variable rates of nano-NPK fertilizers, with and without SA, on fruit yield, WUE and fertilizer use efficiency (FUE) of processing tomatoes, compared to chemical NPK fertilizers alone; and (2) perform a life cycle analysis to compare the economic viability, energy efficiency, and environmental impacts of processing tomato production under a drip-irrigated raised bed plasticulture system.

Materials and Methods

Study site

A replicated field study on drip irrigated plasticulture processing tomato was conducted at South Centers research farm in Piketon, The Ohio State University, Ohio, USA (lat. 39.07° N, long. 83.01° W, elevation 103 m above sea level) during 2022 to 2023 growing seasons.

Long-term average monthly maximum air temperature was recorded at 32.2°C, reaching its peak in August, while the lowest temperature of 15.6°C was observed in September. The mean annual rainfall is 96.2±8.3 cm, with approximately 55% occurring during the crop growing season (May to September). The highest monthly rainfall, measuring 15 cm, was recorded in July [15]. The soil is a poor quality Doles silt loam (*Fine-silty, mixed, active, mesic Aeric Fragiaqualf*), with a pH 6 ± 0.3, organic matter 14.1 ± 4 g kg^{-1}, total nitrogen 1.05 ± 0.24 g kg^{-1}, bulk density 1.28 ± 0.04 g cm^{-3}, sand, silt, and clay 300 ± 40 g kg^{-1}, 55 ± 20 g kg^{-1}, and 150 ± 20 g kg^{-1}, respectively, at 0 to 30 cm depth [15].

Experiment design and cultural practices

A 3 x 2 factorial experiment using a complete randomized design was conducted to evaluate the effects of different levels of nano-NPK fertilization and SA chemigation on processing tomato production under plasticulture-raised bed. Prior to laying the plastic mulch, the field was chisel-plowed, and a basal application of commercial NPK fertilizer (Triple-19) was uniformly applied to all treatment plots at a rate of 20 kg ha^{-1}. The experimental treatments consisted of NPK solutions (Triple 20) applied at equivalent rates of 40, 80, and 120 kg ha^{-1}, each fortified with 1% nano pro—a humic acid and silica-based biostimulant—compared to a control treatment receiving only 120 kg NPK ha^{-1}. Additionally, nano-NPK treatments were combined with SA treatments at two levels: 0% (control) and 0.3% with four replicated plots for each treatment combination. Individual raised bed plots measure 2 m in width and 5 m in length, separated by 50 cm buffer zones. The nano pro (humic acid-based solution with adjuvant) was supplied by Aqua-Yield® (Sandy, UT, USA), and the sodium salicylate (≥ 99% purity) used as the source of SA was obtained from Fisher Scientific® (USA).

Processing tomato seeds (cv. BHN 685) were sown in 72-cell plug trays filled with Metro Mix 360® soilless medium and placed in a controlled growth chamber for germination. Seedlings were transplanted to the field upon reaching a height of 25–30 cm, during the third week of May in both 2022 (May 19) and 2023 (May 21). Plastic-mulched raised beds were prepared with row spacing of 1.6 m and intra-row plant spacing of 60 cm, using a waterwheel transplanter.

Nano-NPK fertilization was administered via drip irrigation over a six-week period, beginning in June and continuing through mid-August in both years. SA was applied twice—once in mid-June and again in early July—coinciding with the peak vegetative growth stage.

Irrigation scheduling was based on 75% of the maximum allowable depletion of available soil moisture and was delivered through drip emitters positioned at a 10 cm soil depth. During treatment applications, all irrigation valves were closed except for the specific line delivering the nano-NPK or SA treatments. Treatments were injected into the pressurized drip system, requiring approximately 15 min for injection, followed by an additional 10 min of clean water irrigation to flush the lines. The header line was then uncapped to drain residual solution between treatments.

All other cultural and plant protection practices, including fungicide applications, were conducted according to the *Midwest Vegetable Production Guide for Commercial Growers*-ID 56 [36].

Tomato yield attributes

Tomato fruits were harvested upon ripening, with harvested fruits graded as marketable or cull (including green), and their respective weights were recorded. Additionally, an average weight of tomatoes and per plant calculation was performed based on randomly selected 50 fresh tomatoes from each replicated plot.

Water- and nutrient-use efficiency

Tomato WUE as well as FUE were calculated [37-38] as follows:

WUE (kg m^{-3}) = Total fruit yield (Mg ha^{-1}) / irrigation applied (m^3 ha^{-1}).

FUE (kg kg^{-1}) = Total fruit yield (Mg ha^{-1}) / NPK fertilizers applied (kg ha^{-1}).

Soil and water analysis

Composite soil (2.54 cm internal diameter) were collected from 0-20 cm depth using a JMC® stainless-steel soil environmental probe fitted with plastic liners before layout of the field experiment. The soil samples were immediately placed in sealable plastic bags and stored at 4°C until further analysis. A portion of the field-moist soil was air-dried under shade at room temperature (~ 25 ^{0}C) for 15 days, ground using an agate mortar and pestle, and passed through a 2 mm sieve prior to analysis.

Soil pH was measured using a glass electrode pH meter (Model 520A, Orion®, Boston, MA, USA). Electrical conductivity was determined in a 1:1 soil-to-distilled water suspension using a conductivity probe. Soil total organic carbon (SOC) and total nitrogen (TN) concentrations were analyzed on finely ground (<125 µm) air-dried samples using a FlashEA 1112 Series CNHS-O dry combustion analyzer®. Bulk density was assessed following the standard core method. Soil particle size distribution was determined using the Bouyoucos hydrometer method, following the oxidation of soil organic matter with 3% hydrogen peroxide and subsequent overnight dispersion in a 10% sodium hexametaphosphate solution. Soil field moisture capacity and permanent wilting point were determined as 0.033 MPa and 1.5 MPa, respectively, using the pressure plate apparatus [39]. Antecedent soil moisture for irrigation scheduling was monitored using a Delta-T soil moisture probe.

Groundwater used for drip irrigation was collected randomly in clean, dry, sterilized plastic bottles. Prior to analysis, samples were filtered through Whatman® No. 42 filter paper. The filtered

water samples were analyzed for pH, electrical conductivity, macro- and micronutrients, and heavy metals using standard analytical protocols [15].

Economic, energy, and environmental assessments

Economic inputs for tomato production were operating costs such as pre-planting and post-planting fertilization, labor, machinery, diesel fuel, agricultural chemicals, sodium salicylate, and NanoPro solution, water, plastic mulching, drip irrigation system, seed, and cash overhead costs and non-cash overhead costs. Economic outputs were the gross revenues, which were calculated by the total product yields (tomato fruits) and market prices in 2022 and 2023. For calculation of economic benefit, the net return, benefit: cost, and economic productivity were determined [29-30] using the following equations:

Net return ($ ha^{-1}) = Economic output − economic input

Benefit: cost ratio = Economic output ÷ economic input

Economic productivity (kg $\$^{-1}$) = Total tomato yield ÷ economic input.

Energy inputs of tomato production include energy from plastic mulching, pre- and post-planting fertilizations, NanoPro solution, sodium salicylate, labor, machinery, diesel fuel, water, drip irrigation system, and seed. Energy outputs of tomatoes consisted of equivalent energy production from fresh tomato fruits. Input and output data were converted into common energy units with the appropriate coefficients of energy equivalence (**Tables S1 – S8**). Energy use efficiency, net energy, and energy productivity were calculated for energy analysis [40-41], which are as follows:

Energy use efficiency or energy rate (%) = (Energy output / energy input) * 100.

Net energy (MJ ha^{-1}) = Energy output − energy input.

Energy productivity (kg MJ^{-1}) = Fresh root, tuber or grain yield ÷ energy input.

Greenhouse gas (GHG) emissions from the tomato production, manufacturing, use and disposing of plastic mulching, machinery, diesel fuel, fertilizers, agricultural chemicals, NanoPro solution, sodium salicylate, and seeds were estimated (**Tables S1 – S8**). GHG emissions including carbon dioxide (CO_2), methane (CH_4), and nitrous oxide (N_2O) were weighed as the GHG emission coefficients (CO_2-equivalent) according to their global warming potential factors of 1, 25, and 298, respectively, following IPCC's 100-year estimates [40, 42]. GHG emissions and GHG intensity of tomato production were calculated [35] as below.

$$\text{Total GHG emission (kg } CO_2 - \text{eq ha}^{-1}) = \sum Q_i \times C_i$$

where Q_i is the quantity of input (i), and C_i indicates the GHG emission coefficients of input (i).

GHG intensity (kg CO_2 ton of tomato^{-1}) = Total GHG emission / Total tomato yield.

Statistical analysis

Multivariate statistical analyses, including a three-way analysis of variance (ANOVA), were performed using Statistical Analysis Software® [43] to evaluate the effects of nano-NPK fertilization and SA levels (as fixed factors) on selected processing tomato response variables. These included total, marketable, and cull fruit yields; WUE- and FUE; as well as economic, energy, and environmental indicators during the 2022 and 2023 growing seasons. Data normality was assessed using the Shapiro–Wilk test, and homogeneity of variances was verified using Levene's test). For all statistical analyses, significant main effects and interactions among predictor variables were separated using the F-protected Duncan's Multiple Range Test at a significance level of $p < 0.05$, unless otherwise specified. All graphical outputs were generated using SigmaPlot®.

Results and Discussion

Tomato yield attributes

Tomato yield components were significantly but variably influenced by nano-NPK fertilization, SA application, and growing seasons, with limited interaction effects among these factors (**Table 1; Fig. 1**). Average fruit yield per plant varied across treatments, with nano-NPK applied at 80 and 120 kg ha^{-1} producing similar or up to 10% higher fruit yields compared to the conventional 120 kg ha^{-1} NPK control. While the average fruit weight remained statistically unchanged, total fruit yield increased by 2% and 10% under nano-NPK fertilization at 80 and 120 kg ha^{-1}, respectively, relative to the control.

Table 1. Effects of different rates of nano-NPK fertilization and salicylic acid (SA) application on tomato fruit attributes in 2022 and 2023 growing seasons (Mean values were presented with standard error of mean).

Year	SA	NPK	Fruit yield	Aver. fruit	Total fruit	Cull fruit	Total fruit:
	(%)	(kg ha^{-1})	(kg plant^{-1})	weight (g)	___ yield (Mg ha^{-1})___		cull fruit

2022			1.7±0.1B$^{\phi}$	118.9±4.2A	39.5±1B	7.7±1.3A	5.1±0.6B
2023			2.6±0.1A	119.6±5.1A	43.4±1.3A	6.6±0.7B	6.6±0.4A
	0		2.1±0.1x$^{\neq}$	117.7±5.8x	39.5±1.1y	7.5±1.5x	5.3±1y
	0.3		2.2±0.1x	120.9±4.9x	43.5±2.1x	6.8±0.8x	6.4±0.8x
		120	2.1±0.1bc$^{\delta}$	121.8±8.7ab	43.8±1.1b	8.2±0.9a	5.3±2.3a
		40NF	1.9±0.1c	115.4±7.4b	29.3±2.3c	7.3±0.6ab	4±3.1ab
		80NF	2.2±0.2ab	118.2±4.9ab	44.6±2.1b	6.9±1.2b	6.5±1b
		120NF	2.3±0.2a	122.3±6.1a	48.2±3.2a	6.3±1.1b	7.6±1.2c
**Nano-NPK x SA x Year interactions**							
2022	0	120	1.7±0.1	122.2±10	40.3±1.4	10.4±3	3.9±0.6
		40NF	1.1±0.1	113.6±7.7	26.3±1.7	7±0.9	3.8±1.9
		80NF	1.7±0.1	116.5±5	39.5±1.9	8.2±1.7	4.8±1.1
		120NF	1.8±0.1	132.9±8.6	42.5±1.9	8±2.1	5.3±0.9
	0.3	120	1.2±0.1	117.5±10.5	45.6±1.1	9.7±1	4.7±1
		40NF	1.9±0.1	119.7±6.9	29±1.7	6.8±1.4	4.3±1.2
		80NF	1.9±0.1	119.2±3.9	45±1.3	6.7±3	6.7±0.4
		120NF	2±0.2	115.4±9.3	48±3.5	5.2±1.2	9.2±3.1
2023	0	120	2.6±0.2	127.8±6.2	43.5±0.8	7.1±1.1	6.1±0.7
		40NF	2.3±0.2	111.1±14.8	28.2±1.1	7.4±1.7	3.8±0.7
		80NF	2.9±0.3	122.9±2.5	46.5±1.6	6.2±1.4	7.5±1.2
		120NF	2.7±0.4	120.2±12.4	48.7±3.1	5.9±0.9	8.2±3.6
	0.3	120	3±0.3	117.3±8	45.8±1.2	12.5±2.1	3.7±1.1
		40NF	2.5±0.6	117.1±15.1	33.8±1.4	8.1±1.5	4.2±0.9
		80NF	2.3±0.1	114.4±9.4	47.4±2.4	6.4±1.3	7.5±1.9
		120NF	2.7+0.2	120.7±10.1	53.5±3.8	6.2±1.8	8.6±2.1

Probability > F					
Nano-NPK x SA	0.124	0.126	0.579	0.904	0.581
Nano-NPK x Year	0.075	0.754	0.037	0.004	0.259
SA x Year	0.127	0.958	0.181	0.157	0.160
Nano-NPK x SA x Year	0.01	0.481	0.21	0.066	0.194

Marketable yield increased significantly—by 6% and 17% under nano-NPK fertilizations of 80 and 120 kg ha^{-1}, respectively—compared to the control (**Fig. 1a**). Additionally, nano-NPK fertilization at these rates significantly reduced cull fruit yield by 11–23% and increased the total yield to cull fruit ratio by 23–43%. These results indicate that nano-NPK fertilization increased marketable yield primarily by reducing the proportion of unmarketable fruits. In contrast, nano-NPK applied at 40 kg ha^{-1} resulted in the lowest total yield, 33% below the control.

Application of SA at 0.3% alone significantly increased total yield by 10%, marketable yield by 15%, and the total yield to cull fruit yield ratio by 21% compared to the control, without significantly affecting other yield traits (**Table 1**). These improvements were also associated with a reduction in the proportion of cull fruits, indicating enhanced fruit quality and yield potential. The effects of nano-NPK and SA on tomato yield parameters were more pronounced in 2023 than in 2022, reflecting inter-annual environmental variability.

Fig. 1. Effects of different rates of nano-NPK fertilization and salicylic acid (SA) application on (**a**) marketable yield of tomato and the regression of (**b**) total- and (**c**) marketable yields on nano-NPK fertilization and SA under drip irrigated plasticulture during 2022 nd 2023 growing seasons (mean values were plotted with standard error).

Regression analyses indicated that nano-NPK applications at 40, 80, and 120 kg ha^{-1}, with and without SA, significantly and non-linearly influenced both total and marketable tomato yields (**Figs. 1b and 1c**). Application of 80 kg ha^{-1} nano-NPK combined with 0.3% SA yielded total fruit outputs comparable to or exceeding those obtained with 120 kg ha^{-1} nano-NPK alone in both years by accounting 97–99% of the variability in total yield (**Fig. 1b**). Similarly, marketable yield exhibited a significant non-linear response to nano-NPK rates (**Fig. 1c**), where 80 kg ha^{-1} nano-

NPK with 0.3% SA achieved yields equal to or greater than those with 120 kg ha^{-1} nano-NPK alone in both 2022 and 2023.

The significant improvements in total and marketable yields, along with reduced cull fruit production under nano-enabled NPK fertilization at 80 and 120 kg ha^{-1}, compared to conventional NPK at 120 kg ha^{-1}, can be attributed to improved nutrient availability, uptake efficiency, and utilization. These factors collectively enhance photosynthesis and assimilate translocation to fruits [14, 16]. Nano-materials, when combined with chemical fertilizers, act as efficient carriers, facilitating nutrient transport through root nanopores, increasing uptake, and boosting metabolic function and yield potential [14-15, 44]. These findings align with previous research demonstrating increased vegetable crop productivity, including in tomatoes, following nano-fortified chemical fertilizer applications [14-15, 26]. Notably, it was observed a 25% increase in marketable tomato yield when Fe nanoparticles replaced chelated Fe in commercial NPK formulations [15].

Furthermore, the application of 0.3% SA significantly enhanced total and marketable yields, as expected, due to its ability to mitigate abiotic and biotic stress, improve plant vigor, and regulate physiological and biochemical processes related to water and nutrient uptake, and fruit development [20, 24, 26, 45].

The lack of statistically significant interaction between nano-NPK and SA may stem from their complementary yet non-linear physiological nad metabolic effects. Although both inputs individually enhance nutrient and water absorption, reactivity, and efficiency, their combined application does not exhibit a simple linear additive effect, likely due to overlapping modes of action. Despite this, the combination of 80 kg ha^{-1} nano-NPK with 0.3% SA produced fruit yields comparable to, or even surpassing, those achieved with 120 kg ha^{-1} of either conventional or nano-NPK alone. This demonstrates that integrated use of SA with reduced nano-fertilizer inputs can optimize yield while potentially lowering production costs and minimizing environmental impacts—supporting a sustainable and efficient fertilization strategy in tomato production systems.

Water- and fertilizer use efficiency of tomato

Tomato WUE was significantly influenced by nano-NPK fertilization, SA application, and growing year, both individually and interactively (**Fig. 2a**). Application of nano-NPK at 80 and 120 kg ha^{-1} significantly increased WUE by 13% to 34% compared to the control, with a more pronounced effect observed in 2023 than in 2022. Similarly, SA applied at 0.3% significantly

improved WUE by 11% relative to the control, again with a stronger response in 2023. Averaged across nano-NPK and SA treatments, WUE varied significantly (by 9%) between years. However, interactions between nano-NPK × SA over time were variable due to significant year effect.

Regression analysis demonstrated a significant, non-linear increase in WUE with nano-NPK application rates (40, 80, and 120 kg ha^{-1}), both with and without SA, across years (**Fig. 2b**). Notably, the combination of 80 kg ha^{-1} nano-NPK with 0.3% SA produced equal or greater yields than 120 kg ha^{-1} nano-NPK without SA, explaining 98% and 87% of the variability in WUE in 2022 and 2023, respectively. When averaged over both years, nano-NPK at 80 or 120 kg ha^{-1} and SA at 0.3%—alone or in combination—consistently matched or exceeded the WUE of the standard 120 kg ha^{-1} NPK treatment.

In contrast to WUE, FUE was also significantly affected by both nano-NPK fertilization and SA application (**Fig. 2c**). Across years, increasing nano-NPK rates led to a decline in FUE from 156% to 74%, although all nano-NPK treatments maintained significantly higher FUE (by 61%) than the conventional 120 kg ha^{-1} NPK control. The effect of nano-NPK on FUE was more evident in 2022 than in 2023. Similarly, SA at 0.3% increased FUE by 23% on average, with a more pronounced effect in 2022 (by 37%) compared to 2023 (by 10%). While the year alone did not significantly influence FUE, a significant nano-NPK × SA × year interaction was detected.

Fig. 2. Effects of nano-NPK fertilization and salicylic acid (SA) application on (a) water- and (b) fertilizer-use efficiency processing tomato and the regression of (c) water- and (d) fertilizer-use efficiency on nano-NPK fertilization and SA application under drip irrigated plasticulture during 2022 nd 2023 growing seasons (mean values were plotted with standard error).

Regression analysis indicated that FUE declined significantly and non-linearly with increasing nano-NPK rates (**Fig. 2d**). However, application of 80 kg ha^{-1} nano-NPK with 0.3% SA achieved similar or higher marketable fruit yields compared to 120 kg ha^{-1} nano-NPK alone, in both 2022 and 2023. Combined across years, treatments involving 120 kg ha^{-1} nano-NPK and/or 0.3% SA yielded FUE outcomes equal to or better than the conventional NPK control.

The enhanced WUE and optimal FUE in nano-NPK fertilizer especially at 80 kg ha^{-1} treatments are attributed to improved nutrient and water uptake, facilitated by the nanoporous structure of plant roots that enhances the rapid absorption and translocation of nutrient-bound

nanomaterials [7, 14-15]. However, diminishing returns in FUE at higher nano-NPK rates likely resulted from fertilizer use inefficiencies, with excess nutrients not contributing to yield increases [46]. Previous studies also reported that while traditional chemical fertilization provides higher crop yield and income stability, excessive chemicals application does not produce a higher yield but can pollute the environment [26, 46-47].

Despite some interaction effects, the combined application of 80 kg ha^{-1} of nano-NPK fertilization with 0.3% SA emerged as the most effective strategy for optimizing both WUE and FUE in tomato production. These findings highlight a promising, cost-effective, and environmentally sustainable alternative to conventional high-input NPK fertilization practices.

Economic viability of tomato production

The average estimations of total economic inputs (total cost), total economic outputs (gross revenues), benefit: cost ratio, and net income for tomato production with different rates of nano-NPK fertilization and SA chemigation were illustrated (**Table S1-S8**). The estimated input cost per hectare were $ 7094 for the control (120 kg NPK alone), $7120 for the 120 kg nano-NPK, $7077 for the 120 kg NPK with SA chemigation, and $7114 for the 120 kg nano-NPK with SA chemigation. This showed a negligible increase in production costs with the nano-NPK with or without SA chemigation compared to conventional NPK fertilization. However, the input costs were $6757 for the 80 kg nano-NPK fertilization with and without SA chemigation. In contrast to total input costs, the estimated economic outputs per hectare were $7920 for the control (120 kg NPK alone), $8128 and $8619 for the 80 and 120 kg nano-NPK, $8637 for the 120 kg NPK with SA chemigation, and $8770 and $9592 for the 80 and 120 kg nano-NPK with SA chemigation, respectively. This indicates that tomatoes fertilized with 80 and 120 kg nano-NPK could yield $208 and $ 699 as profits than the control, while 120 kg NPK with SA yield $717 profit, and 80 and 120 kg nano-NPK with SA chemigation yield higher profits ($850 and $1672) than the control (120 kg NPK).

Fig. 3. Effects of nano-NPK fertilization and salicylic acid (SA) application on water- and fertilizer-use efficiency processing tomato under drip irrigated plasticulture during 2022 nd 2023 growing seasons (mean values were plotted with standard error).

Total economic cost, including inputs and overheads of tomato production in response to nano-NPK and SA application except 40 kg NPK/ha with or without SA, was lower than the total economic outputs. As a result, average tomato production was significantly profitable in both years, with the highest net return of \$2478 ± \$523 and \$2012 ± \$265 in 120 and 80 kg nano-NPK ha^{-1} with SA, followed by \$1560 ± \$19 in 120 kg NPK with SA ha^{-1}, \$1498 ± \$587 in 120 nano-NPK ha^{-1} compared to \$825 ± \$303 in the control (120 kg NPK ha^{-1}). However, the net return was negative (\$503 ± \$455 and \$1303 ± \$663) in 40 kg nano-NPK with or without SA, when compared to the control. The effect of nano-NPK or SA and their interaction on net return of tomato production was higher in 2023 than in 2022 (**Fig. 3a**).

The benefit: cost ratio was significantly higher in both nano-NPK fertilization and SA application; however, the highest benefit: cost ratio (1.5 ± 0.1) was observed in 120 and 80 kg nano-NPK with SA compared to 1.3 ± 0.05 in the control (**Fig. 3b**). The effect of nano-NPK or SA and their interaction on benefit: cost ratio of tomato production was higher in 2023 than in 2022. Likewise, the economic productivity was higher with both nano-NPK and SA application when compared to the control. When nano-NPK applied at 80 and 120 kg ha^{-1}, the economic productivity of tomatoes had 6.3 and 6.4 kg \$$^{-1}$ (increase by 7 to 8%), the application of SA had the economic productivity of 6.5 kg \$$^{-1}$ (increase by 10%) compared to the control (**Fig. 3c**). However, nano-NPK fertilization at 80 and 120 kg^{-1} with SA application had more economic tomato productivity of 6.9 and 7.1 kg \$$^{-1}$ (increase by 17 and 20%), respectively, than that of the control. The nano-NPK and SA had a higher economic tomato productivity in 2023 than in 2022.

Despite minimal differences in input costs, gross economic outputs increased notably with nano-NPK and SA application. Compared to the control (\$7920), revenues improved with 80 kg (\$8128) and 120 kg (\$8619) nano-NPK ha^{-1}, and with 120 kg NPK with SA (\$8637). The highest outputs were achieved with 80 and 120 kg nano-NPK with SA (\$8770 and \$9592), reflecting a synergistic effect on tomato yield [48-49].

Net income followed similar trends, with the greatest returns from 120 and 80 kg nano-NPK + SA (\$2478 ± 523 and \$2012 ± 265), significantly higher than the control (\$825 ± 303). In contrast, 40 kg nano-NPK treatments produced negative returns, indicating insufficient fertilization. The benefit: cost ratio peaked at 1.5 ± 0.1 for nano-NPK + SA, compared to 1.3 ± 0.05 in the control, aligning with reports that nano-fertilizers improve economic returns through enhanced nutrient-use efficiency [50-51]. Economic productivity (kg \$$^{-1}$) also improved: from 6.0

in the control to 6.3–6.4 with nano-NPK, 6.5 with SA, and up to 7.1 with combined treatments—an increase of 20% [52].

Higher returns and benefit: cost ratios in 2023 suggest favorable environmental conditions and improved input synergy over time. Given its high market value and input responsiveness, tomato production—especially using 80–120 kg nano-NPK with 0.3% SA—is economically superior to conventional methods and traditional crops [53].

Energy efficiency of tomato production

Tomato production using nano-NPK fertilizers and SA amendments demonstrated a trade-off between energy input and output across all treatments (**Fig. 4**). Total energy input consistently exceeded energy output, leading to net negative energy use, which is characteristic of high-input intensive horticultural systems [54]. Among input components, plastic mulch accounted for the largest share of energy consumption, followed by NPK fertilizers, diesel fuel, and herbicides, which aligns with findings in similar energy audits for vegetable cropping systems [55-56].

Despite the overall negative energy balance, nano-NPK fertilization, particularly at 80 and 120 kg ha^{-1} rates, significantly reduced net negative energy use compared to the conventional control (**Fig. 4a**). The combined application of nano-NPK with 0.3% SA further enhanced this reduction, with the most effective treatment (80 kg ha^{-1} + SA) decreasing net energy use by 9611 MJ ha^{-1}. This improvement is attributable to enhanced nutrient use efficiency and reduced losses typically associated with nano-fertilizer technology.

The energy use efficiency (EUE), a critical metric of sustainability, improved with nano-NPK and SA applications (**Fig. 4b**). Treatments with nano-NPK alone increased EUE by 3.7–4.6%, while integration with SA achieved an 8.3–8.9% improvement relative to the control. SA alone also increased EUE by 4.4%, likely due to its role in enhancing plant physiological responses and resource use. These findings support previous studies that highlight the synergistic effects of biostimulants and nano-fertilizers on crop performance and resource optimization [57].

The energy productivity (EP), representing the crop yield per unit energy input, exhibited a non-linear decline with increasing nano-NPK levels, yet all nano-NPK treatments had higher EP than the control (**Fig. 4c**). Notably, 80 kg ha^{-1} nano-NPK with or without SA optimized energy productivity (0.57 kg MJ^{-1}) compared to the control (0.52 kg MJ^{-1}), indicating a more efficient energy investment per yield unit. These outcomes suggest that moderate nano-NPK fertilization,

especially when combined with SA, presents a viable approach to improve the energy sustainability of tomato production systems.

Fig. 4. Effects of nano-NPK fertilization and salicylic acid (SA) on (**a**) net energy use, (**b**) energy-use efficiency, and (**c**) energy productivity of processing tomato under drip irrigated plasticulture during 2022 nd 2023 growing seasons (mean values were plotted with standard error).

Environmental compatibility of tomato production

The results showed that total GHG emissions decreased with nano-NPK fertilization compared to the conventional NPK treatment. However, increasing the rate of nano-NPK fertilization, particularly at higher doses with or without SA application, did not further reduce emissions; in some cases, it maintained or slightly increased GHG emissions (**Table S1–S8**). The application of SA alone did not have a significant influence on total GHG emissions, likely due to the minimal volume required for SA treatment.

Fig. 5. Effects of nano-NPK fertilization and salicylic acid (SA) on greenhouse gas ratio or intensity of processing tomato and the regression of (c) water- and (d) fertilizer-use efficiency on nano-NPK fertilization and SA application under drip irrigated plasticulture during 2022 nd 2023 growing seasons (mean values were plotted with standard error).

When considering GHG emission intensity (i.e., the GHG ratio expressed as kg GHG emitted per ton of tomato production), the lowest values were observed with nano-NPK fertilization at 80 and 120 kg ha^{-1}, suggesting improved environmental efficiency of tomato production under these treatments (**Fig. 5; Table S1–S8**). This indicates that while total GHG emissions may remain similar or slightly increase with higher nano-NPK application, the emissions per unit of yield decrease—an important metric in sustainable crop production. Additionally, the SA application contributed to lowering the GHG ratio compared to the control, although the interaction between nano-NPK and SA was not statistically significant.

Notably, growing season played a significant role, with stronger effects of nano-NPK and SA observed in 2022 compared to 2023. The interaction between year and nano-NPK or SA treatments influenced the GHG ratio, emphasizing the potential impact of environmental variability and weather patterns on treatment efficacy.

The changes in total GHG emissions across treatments are primarily attributed to the quantity of NPK fertilizer applied, consistent with previous findings that link fertilizer input—particularly nitrogen—as a major driver of GHG emissions in agricultural systems [58]. Other studies emphasized that optimizing inputs such as diesel fuel and nitrogen could substantially reduce GHG emissions [28, 59].

Overall, the results underscore the importance of precision nutrient management in reducing the environmental footprint of crop production. Nano-fertilizers, when applied at optimized rates (e.g., 80–120 kg ha^{-1}), offer a promising strategy to maintain or even increase tomato yields while minimizing GHG emissions. This supports the broader conclusion that avoiding excessive use of chemical fertilizers can prevent trade-offs between high crop yield and environmental sustainability [17, 28, 59].

Conclusions

Application of nano-NPK at 80 and 120 kg ha^{-1} significantly increased total and marketable tomato yields by up to 10% and 17%, respectively, mainly by reducing cull fruit by 11–23%. The 40 kg ha^{-1} treatment underperformed. SA alone improved yields by 10–15%, and its combination with 80 kg ha^{-1} nano-NPK matched or exceeded the 120 kg ha^{-1} treatment, suggesting potential input savings. WUE rose by 13–34% with nano-NPK and an additional 11% with SA, especially in 2023. Although FUE declined at higher rates, it remained above the control, with SA boosting it by 23%. Regression analysis identified 80 kg ha^{-1} nano-NPK + SA as the most efficient. This treatment also achieved the highest economic returns (up to $2,478 ha^{-1}), benefit: cost ratio (1.5), and energy-use efficiency (+8.9%). Nano-NPK, particularly with SA, also reduced GHG emissions and intensity, supporting its potential as a scalable, sustainable tomato production strategy.

Acknowledgements

We acknowledged the financial support provided by the Ohio Department of Agriculture via USDA Specialty Crops Block grant (2021-2024) to conduct the research. We are thankful to Professor Gary Gao for allowing us to use his laboratory facilities and Thomas Harker, Research Associate and Wayne Lewis, Farm manager, for their continuous support to planting, monitoring, data collection, and harvesting tomatoes at OSU South Centers.

Author Contributions

Conceptualization: Khandakar Rafiq Islam

Data curation: Zhenhao Guan, Mohammad Arifur Rahman, Khandakar Rafiq Islam

Formal Analysis: Zhenhao Guan, Mohammad Arifur Rahman, Khandakar Rafiq Islam

Funding acquisition: Khandakar Rafiq Islam, Mohammad Arifur Rahman,

Investigation: Zhenhao Guan, Khandakar Rafiq Islam, Mohammad Arifur Rahman,

Methodology: Zhenhao Guan, Khandakar Rafiq Islam, Mohammad Arifur Rahman

Project administration: Khandakar Rafiq Islam

Resources: Khandakar Rafiq Islam, Mohammad Arifur Rahman

Software: Khandakar Rafiq Islam, Mohammad Arifur Rahman

Supervision: Khandakar Rafiq Islam, Mohammad Arifur Rahman

Validation: Khandakar Rafiq Islam, Mohammad Arifur Rahman

Visualization: Khandakar Rafiq Islam, Mohammad Arifur Rahman,

Writing-original draft: Khandakar Rafiq Islam, Mohammad Arifur Rahman.

Writing-review & editing: Khandakar Rafiq Islam.

References

1. FAOSTAT. Statistics Division of the Food and Agriculture Organization of the United Nations. World Food and Agriculture – Statistical Yearbook. Rome. https://doi.org/10.4060/cc8166en.; 2023.
2. Martí R, Roselló S, Cebolla-Cornejo J. Tomato as a source of carotenoids and polyphenols targeted to cancer prevention. Cancers (Basel). 2016; 8: 58. https://doi.org/10.3390/cancers8060058.
3. Rahman MA, Harker T, Lewis W, Islam KR. Chelated- and nano iron fertilization affects nutrient uptake and translocation in fresh market tomatoes. Journal of Plant Nutrition. 2024; 47: 1152-1174. https://doi.org/10.1080/01904167.2024.2304166.
4. Shewta S, Sood A. A review on nanotechnological advancements in tomato: a model plant. The Journal of Horticultural Science and Biotechnology. 2023; 98: 563–579. https://doi.org/10.1080/14620316.2023.2183903.
5. Zhang Y, Wang H, Lei Q, Luo J, Lindsey S, Zhang J, Zhai L, Wu S, Zhang J, Liu X, Ren T, Liu H. Optimizing the nitrogen application rate for maize and wheat based on yield and

environment on the Northern China Plain. Science of the Total Environment. 2018; 618: 1173–1183.

6. Zhou R, Kong L, Wu Z, Rosenqvist E, Wang Y, Zhao L, Zhao T, Ottosen CO. Physiological response of tomatoes at drought, heat and their combination followed by recovery. Physiologia Plantarum. 2019; 165: 144–154. https://doi.org/10.1111/ppl.12764.

7. Zhang H, Tan C, Wang X, Zhao L, Li Y. Effects of different irrigation and nitrogen levels on tomato growth, yield, WUE, and NUE under drip-fertigated conditions. Agronomy. 2023; 13: 1068. https://doi.org/10.3390/agronomy13051068.

8. Bratovcic A, Hikal W, Said-Al Ahl H, Tkachenko K, Baeshen R, Sabra A, Sany H. Nanopesticides and nanofertilizers and agricultural development: scopes, advances and applications. Open Journal of Ecology. 2021; 11: 301–316. https://doi.org/10.4236/oje.2021.114022.

9. Aqeel U, Aftab T, Khan MMA, Naeem M, Khan MN. A comprehensive review of the impacts of diverse nanoparticles on growth, development and physiological adjustments in plants under changing environment. Chemosphere. 2022; 291: 132672. https://doi.org/10.1016/j.chemosphere.2021.132672 PMID: 34756946.

10. Malica MA, Joshi V, Rajasekhar M, Mallesh S, Sathish G. Effect of nano fertilizers on growth and yield of tomato (*Solanum lycopersicum* L.). International J. Research in Agronomy. 2024; 7: 163-168. https://doi.org/10.33545/2618060X.2024.v7.i10c.1714.

11. Berbezier I, De Crescenzi M. Self-assembly of nanostructures and nanomaterials. Beilstein Journal of Nanotechnology. 2015: 6: 1397–1398. https://doi.org/10.3762/bjnano.6.144.

12. Baŕzana G, Garcıa-Gómez P, Carvajal M. Nanomaterials in plant systems: Smart advances related to water uptake and transport involving aquaporins. Plant nano biology. 2022; 1: 100005–100005. https://doi.org/10.1016/j.plana.2022.100005.

13. Abdel-Aziz HMM, Hasaneen MNA, Omer AM. Impact of engineered nanomaterials either alone or loaded with NPK on growth and productivity of French bean plants: seed priming vs foliar application. South African Journal of Botany. 2019; 125:102–108. https://doi.org/10.1016/j.sajb.2019.07.005.

14. El-Desouky HS, Islam KR, Bergefurd B, Gao G, Harker T, Abd-El-Dayem H, et al. Nano iron fertilization significantly increases tomato yield by increasing plants' vegetable growth and photosynthetic efficiency. Journal of Plant Nutrition. 2021; 1–15. https://doi.org/10.1080/01904167.2021.1871749.

15. Rahman MA, Harker T, Lewis W, Islam KR. Nano and chelated iron fertilization influences marketable yield, phytochemical properties, and antioxidant capacity of tomatoes. PLoS ONE. 2023; 18: e0294033. https://doi.org/10.1371/journal.pone.0294033.

16. Alam P, Yalcin M, Faizan M, Albalawi T. Response of tomato to silicon dioxide nanoparticles under salinity: Impact on photosynthesis, antioxidant enzymes activity, stress biomarkers and osmoregulatory substances. Plant Nano Biology. 2025; 13: 100171, ISSN 2773-1111, https://doi.org/10.1016/j.plana.2025.100171.

17. Zhang X, Davidson EA, Mauzerall DL, Searchinger TD, Dumas P, Shen Y. Managing nitrogen for sustainable development. Nature. 2015; 528: 51–59. https://doi.org/10.1038/nature15743.

18. Chen J, Liu X, Zheng J, Zhang B, Lu H, Liu X, Zhu Q. Effects of nano-fertilizer on yield and quality of greenhouse tomatoes. Journal of Plant Nutrition and Soil Science. 2018; 181: 784-792.

19. Hayat S, Ahmad A, Nasser MA. Salicylic Acid - Plant Growth and Development. Springer. ISBN 978-94-007-6427-9. DOI 10.1007/978-94-007-6428-6.; 2013.

20. Khan MIR, Fatma M, Per TS, Anjum NA, Khan NA. Salicylic acid-induced abiotic stress tolerance and underlying mechanisms in plants. Frontiers in Plant Science. 2015; 6: 462.

21. Wani AB, Chadar H, Wani AH, Singh S, Upadhyay N. Salicylic acid to decrease plant stress. Environmental Chemistry Letters. 2016; 15: 101–123. https://doi.org/10.1007/s10311-016-0584-0.

22. Srinivasa C, Umesha S, Pradeep S, Ramu R, Gopinath SM, Ansari MA, Alomary MN, Ali A, Ahmad W, Kollur SP, Shivamallu C. Salicylic acid-mediated enhancement of resistance in tomato plants against *Xanthomonas perforans*. Saudi Journal of Biological Science. 2022; 29: 2253-2261. doi: 10.1016/j.sjbs.2021.11.067.

23. Irkitbay A, Sapahkova Z, Didenko N, Madenova A, Kanat G, Islam KR. Evaluating salicylic acid and oxalic acid on the enzyme activities, yield and stress tolerance in wheat (*Triticum aestivum* L.). Research on Crops. 2023; 24: 219–228. https://doi.org/10.31830/2348-7542.2023.ROC-926.

24. Jia I, Jiang Z. Salicylic acid alleviates salt stress in soybean by improving anti-oxidant capacity and growth performance. Pakistan Journal of Botany. 2023; 55: 37-44. http://dx.doi.org/10.30848/PJB2023-1(31)

25. Souri MK, Tohidloo G. Effectiveness of different methods of salicylic acid application on growth characteristics of tomato seedlings under salinity. Chemical and Biology Technologies in Agriculture. 2019; 6: 26. https://doi.org/10.1186/s40538-019-0169-9.

26. Rahman A, Zhenhao G, Islam KR, Brown E. Enhancing tomato phytochemicals and fruit quality through nano-NPK and salicylic acid chemigation. Scientia Horticulturae. 2025; 342: 114048, ISSN 0304-4238, https://doi.org/10.1016/j.scienta.2025.114048.

27. Abdel-Wahab MM, El-attar AB, Mahmoud AA. Economic evaluation of nano and organic fertilizers as an alternative source to chemical fertilizers on Carum carvi L. plant yield and components. Agriculture (Polnohospodarstvo). 2017; 63: 33-49.

28. Hosseinzadeh-Bandbafha H, Nabavipelesaraei A, Khanali, M, Ghahderijani, M, Chau K. Application of data envelopment analysis approach for optimization of energy use and reduction of greenhouse gas emission in peanut production of Iran. Journal of Cleaner Production. 2018; 172: 1327–1335

29. Aegerter B, Lazicki P, Miyao G, Stewart D, Murdock J, Goodrich B. Sample costs to produce processing tomatoes sub-surface, drip irrigated (SDI) in the Sacramento valley & northern delta. 2023; University of California Agriculture and Natural Resources Cooperative Extension, UC Davis Department of Agricultural and Resource Economics.

30. Fang YR, Liu JA, Steinberger, Y, Xie GH. Energy use efficiency and economic feasibility of Jerusalem artichoke production on arid and coastal saline lands. Industrial Crops Production. 2018: 117: 131–139.

31. Stolarski MJ, Krzyaniak M, Tworkowski J, Zauski D, Szczukowski S. Camelina and crambe production—Energy efficiency indices depending on nitrogen fertilizer application. Industrial Crops Production. 2019; 137: 386–395.

32. Zhang W, Qian C, Carlson KM, Ge X, Wang X, Chen X. Increasing farm size to improve energy use efficiency and sustainability in maize production. Food and Energy Security. 2021; 10: e271.

33. Chen X, Xu X, Lu Z, Zhang W, Yang J, Hou Y, Wang X, Zhou S, Li Y, Wu L, et al. Carbon footprint of a typical pomelo production region in China based on farm survey data. Journal of Cleaner Production. 2020; 277: 124041.

34. Xiong L, Liang C, Ma B, Shah F, Wu W. Carbon footprint and yield performance assessment under plastic film mulching for winter wheat production. Journal of Cleaner Production. 2020; 270: 122468.

35. Tang C, Lu Y, Jiang B, Chen J, Mo X, Yang Y, Wang Z. Energy, economic, and environmental assessment of sweet potato production on plantations of various sizes in South China. Agronomy. 2022; 12: 1290. https://doi.org/10.3390/agronomy12061290.

36. Egel D. The Midwest Vegetable Production Guide For Commercial Growers 2021 (ID-56). Issue 684. Vegetable Crops Hotline. Purdue University. https://vegcropshotline.org/article/the-midwest-vegetable-production-guide-forcommercial-growers-2021-id-56.; 2020.

37. Kang S, Shi W, Zhang J. An improved water-use efficiency for maize grown under regulated deficit irrigation. Field Crops Research. 2000; 67: 207 - 214.

38. Lavrenko SO, Lavrenko NM, Maksymov DO, Maksymov MV, Didenko NO, Islam KR. Variable tillage depth and chemical fertilization impact on irrigated common beans and soil physical properties. Soil and Tillage Research 2021; 212: 105024, https://doi.org/10.1016/j.still.2021.105024.

39. Klute A. Water Retention: Laboratory Methods, Part 1: Physical and Mineralogical Methods. In: Methods of Soil Analysis (Klute A, ed.), pp. 635–662. No. 9, Madison, WI. USA: Soil Science Society of America, Inc. Publisher.; 1986.

40. Ntinas GK, Neumair M, Tsadilas CD, Meyer J. Carbon footprint and cumulative energy demand of greenhouse and open-field tomato cultivation systems under Southern and Central European climatic conditions. Journal of Cleaner Production. 2017; 142: 3617-3626.

41. Deng J, Ni H, Zhang Z, Usman S, Yang X, Shen Y, Li Y. Designing productive, energy-efficient, and environmentally friendly production systems by replacing fallow period with annual forage cultivation on the Loess Plateau of China. Journal of Cleaner Production. 2021; 320: 128660.

42. Eggleston S, Buendia L, Miwa K, Ngara T, Kiyoto T. IPCC Guidelines for National Greenhouse Gas Inventories; Institute for Global Environmental Strategies (IGES) for the IPCC: Hayama, Japan, 2006.

43. SAS Institute. SAS Statistics Users' Guide R. 9.4. SAS Institute. Cary, NC, USA.; 2023.

44. Kah M, Kookana RS, Gogos A, Bucheli TD. A critical evaluation of nanopesticides and nanofertilizers against their conventional analogues. Nature Nanotechnology. 2018; 13: 677–684. https://doi.org/10.1038/s41565-018-0131-1 PMID: 29736032.

45. Rivas-San Vicente M, Plasencia J. Salicylic acid beyond defense: its role in plant growth and development. Journal of Experimental Botany. 2011; 62: 3321-3338.

46. Helal MID, El-Mogy MM, Khater HA, Fathy MA, Ibrahim FE, Li YC, Tong Z, Abdelgawad KF. A controlled-release nanofertilizer improves tomato growth and minimizes nitrogen consumption. Plants. 2023; 12: 1978. https://doi.org/10.3390/plants12101978.

47. Ju XT, Xing GX, Chen XP, Zhang SL, Zhang LJ, Liu X,J, Cui, ZL, Yin B, Christie P, Zhu ZL, et al. Reducing environmental risk by improving N management in intensive Chinese agricultural systems. Proceedings of the. National Academy of Sciences, USA. 2009: 106: 3041–3046.

48. Mahil E, Kumar S. Nano-fertilizers and their smart delivery system. In: S. Rakshit et al. (Eds.), Nanotechnology: Potentials for Improving Crop Productivity (pp. 119-137). Springer. https://doi.org/10.1007/978-981-13-5862-3_7; 2019.

49. Kaya C, Ashraf M, Alyemeni MN, Ahmad P. Responses of tomato plants to salicylic acid and nano-silicon under high temperature stress. Botany Letters. 2021; 168: 231-241. https://doi.org/10.1080/23818107.2020.1870260.

50. Saberioon MM, Amin MSM, Aimrun W, Gholizadeh A. Economic and agronomic impacts of precision nutrient management. Precision Agriculture. 2020; 21: 1131–1155. https://doi.org/10.1007/s11119-020-09721-5.

51. Raliya R, Saharan V, Dimkpa C, Biswas P. Nanofertilizer for precision and sustainable agriculture: Current state and future perspectives. Journal of Agricultural and Food Chemistry. 2016; 66: 6487–6503. https://doi.org/10.1021/acs.jafc.7b02178.

52. Dimkpa CO, Bindraban PS. Fortification of micronutrients for efficient agronomic production: a review. Agronomy for Sustainable Development. 2016; 36: 7. https://doi.org/10.1007/s13593-015-0346-6.

53. Khokhar MB, Khokhar BA, Ali S. An economic analysis of tomato production under tunnel farming in Punjab, Pakistan. Sarhad Journal of Agriculture. 2021; 37: 98-105. https://doi.org/10.17582/journal.sja/2021/37.1.98.105.

54. Esengun K, Erdal G, Gündüz O, Erdal H. An economic analysis and energy use in stake-tomato production in Tokat province of Turkey. Renewable Energy. 2007; 32: 1873–1881.

55. Yilmaz I, Akcaoz H, Ozkan B. An analysis of energy use and input costs for cotton production in Turkey. Renewable Energy. 2005; 30: 145–155.

56. Ozkan B, Ceylan RF, Kizilay H. Energy inputs and crop yield relationships in greenhouse winter crop tomato production. Renewable Energy. 2011; 36: 3217–3221.

57. Ramesh NK, Monahar PD, Veena J, Padmavathamma AS and Syamraj NC. Effect of antioxidants on shelf life and quality minimally processed pomegranate arils cv. Bhagwa. Advances in Life Science. 2016; 5: 4678–4682.

58. Snyder CS, Bruulsema TW, Jensen TL, Fixen PE. Review of greenhouse gas emissions from crop production systems and fertilizer management effects. Agriculture, Ecosystems & Environment. 2009; 133: 247-266.

59. Rezvani MP, Feizi H, Mondani F. Evaluation of tomato production systems in terms of energy use efficiency and economical analysis in Iran. Notulae Scientia Biologicae. 2011; 3: 58-65. https://doi.org/10.15835/nsb346279.

Manuscript Click here to access/download;Figure;Fig-1.tif

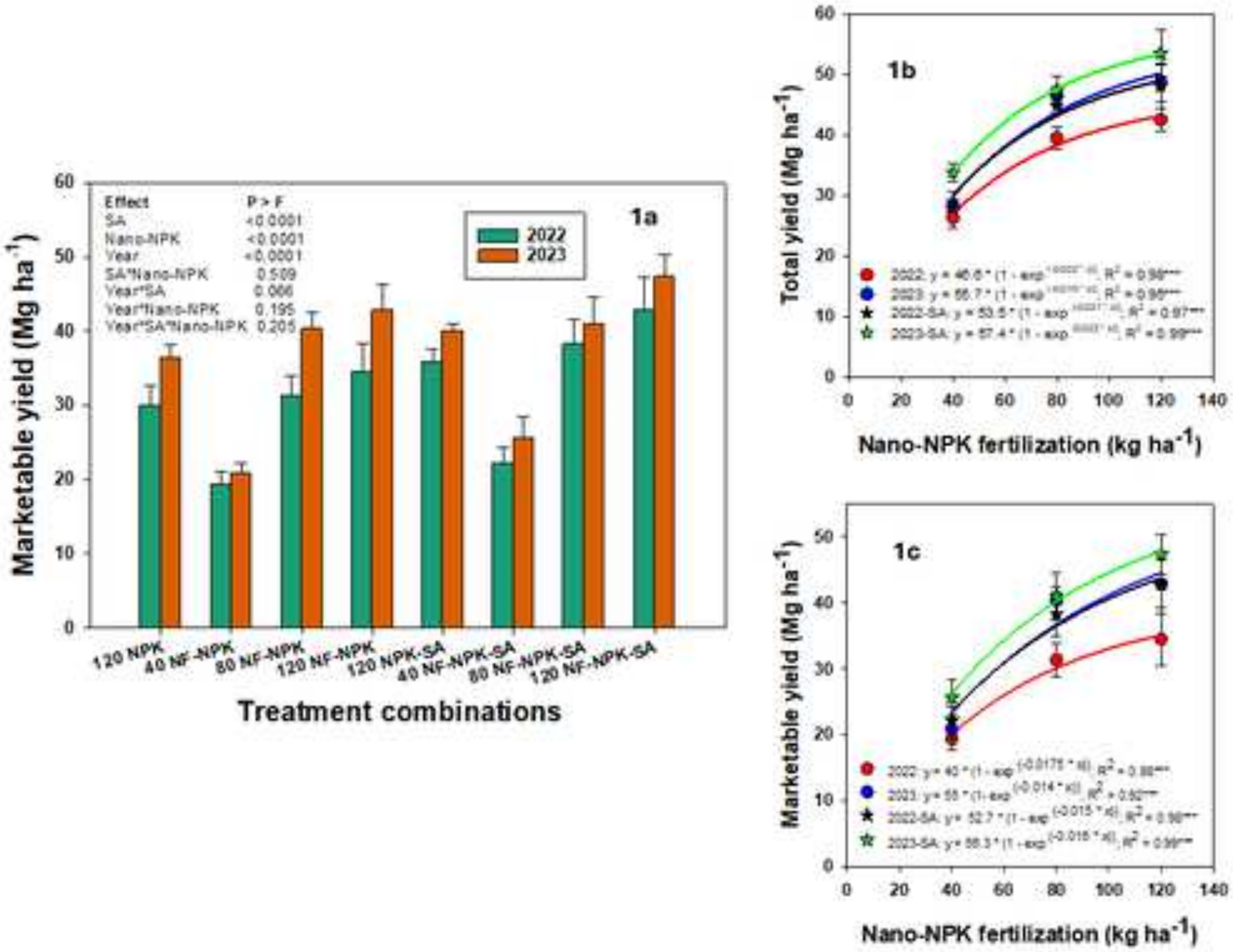

Manuscript

Click here to access/download;Figure;Fig-2.tif

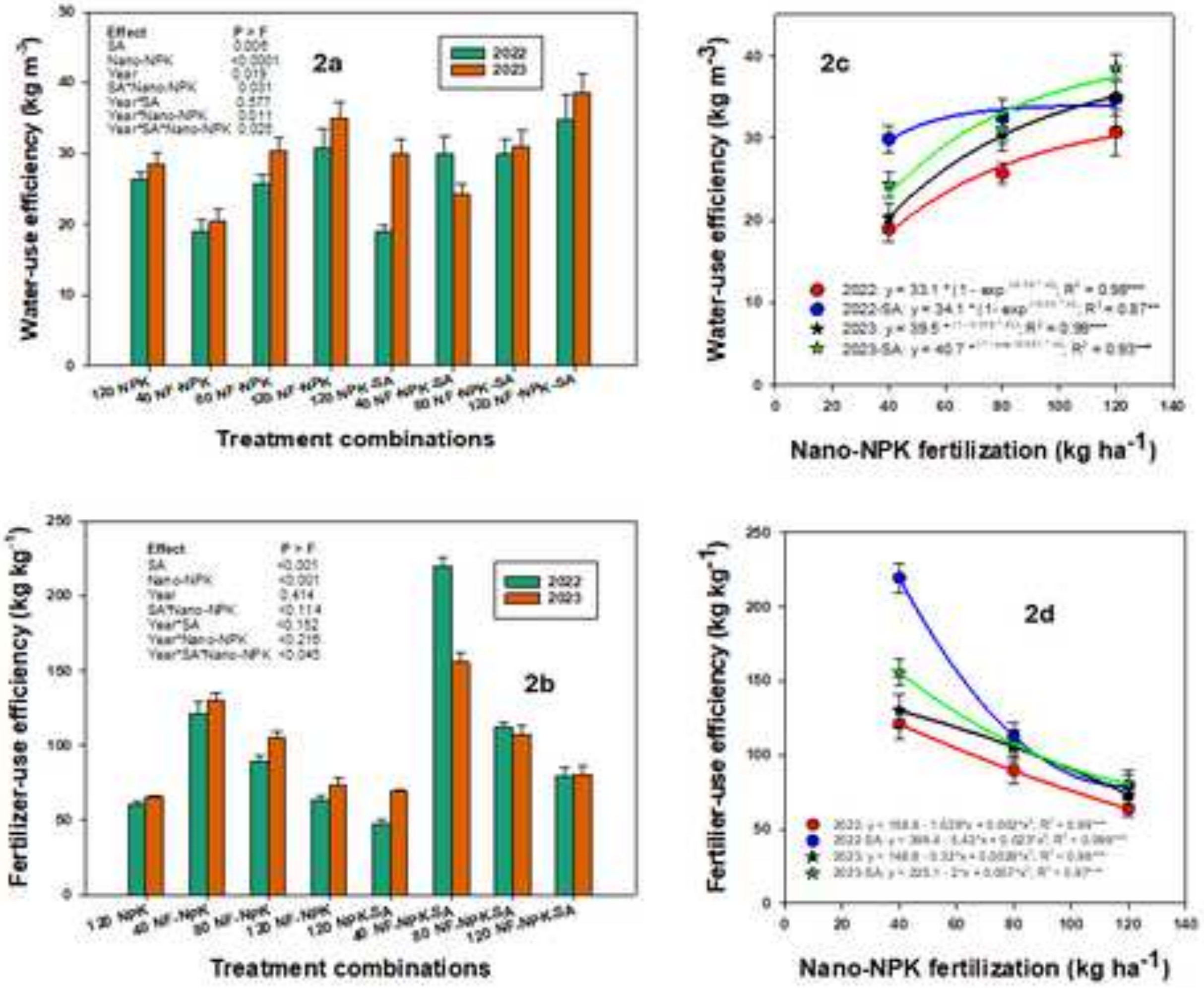

Manuscript Click here to access/download;Figure;Fig-3.tif

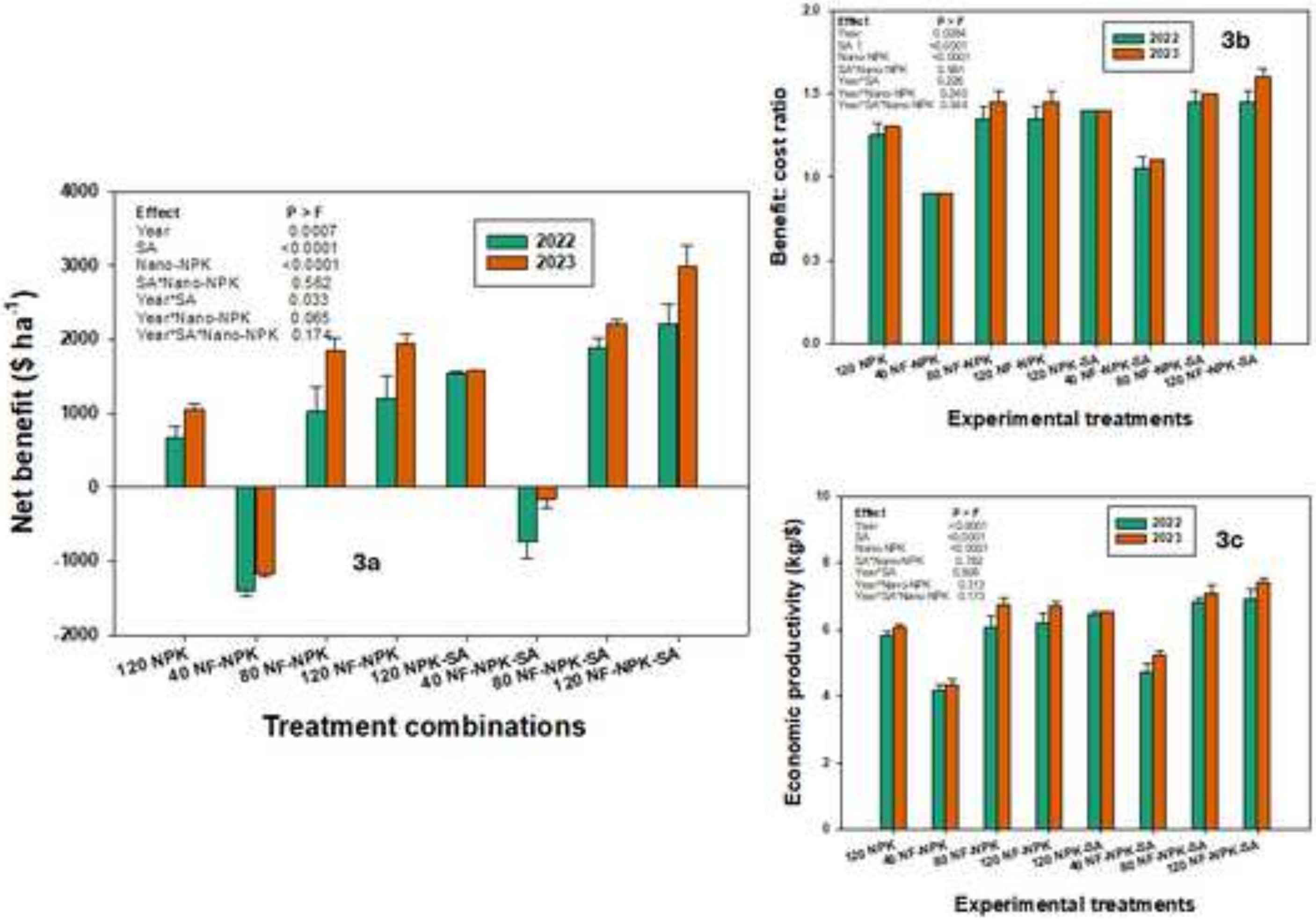

Manuscript

Click here to access/download;Figure;Fig-4.tif

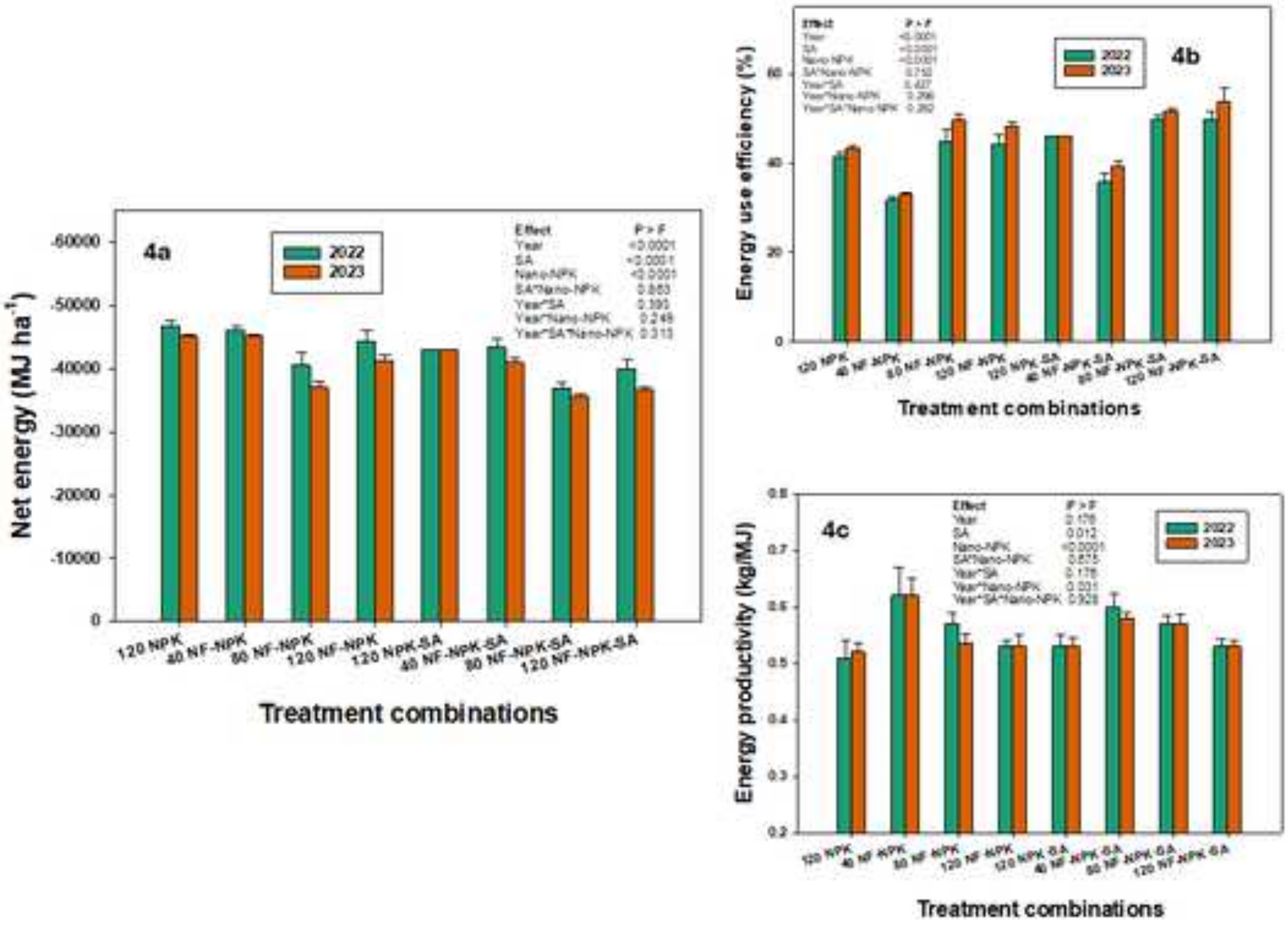

Click here to access/download;Figure;Fig-5.tif

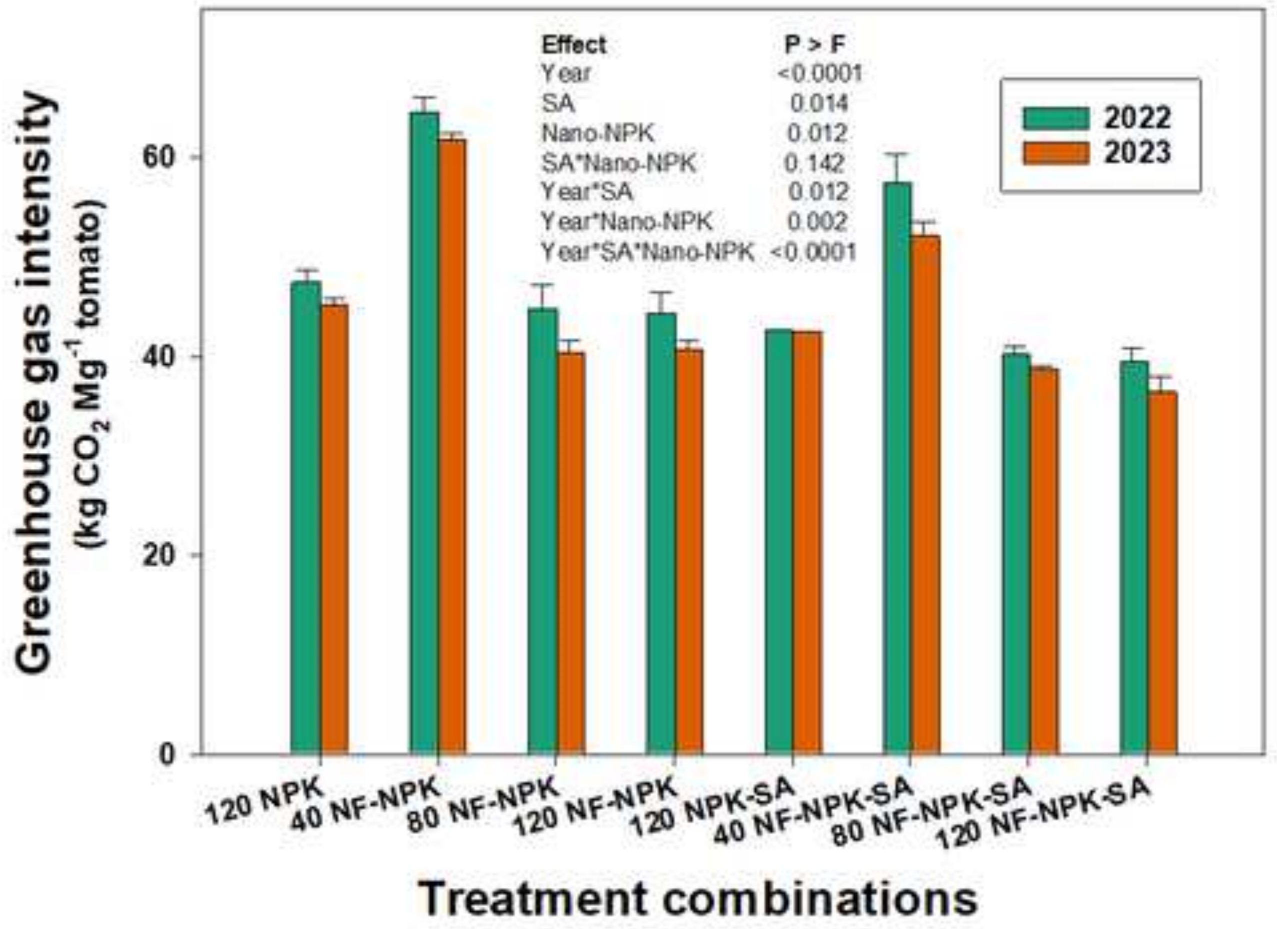

Manuscript

Click here to access/download
Supporting Information
Supporting Information.docx
