# Supplementary material for: Improving tomato yield and production economics with Nano-NPK fertilization and salicylic acid chemigation
Source: PLoS One. 2026 May 4;21(5):e0348207. doi: 10.1371/journal.pone.0348207 (PMC13138637; doi:10.1371/journal.pone.0348207)
Supplement: Attachment — Submitted filename: comments.docx [file pone.0348207.s003.docx]

In the abstract, specify which type of NPK fertilizer was used.

Keywords should be arranged alphabetically.

Line 68: the end of paragraph you should use reference.

Line 148: for soil characteristics you used reference, while you should do all experiment about it. It should be mentioned how these parameters mesueared?

Line 154: all characteristics of NPK need. Please write where NPK purchased? Which brand? And all-important characteristics.

Line 165: please write more about the condition of growth chamber like temp, RH, LIGTH and …

Line 186: according which reference?

In this experiment 4 replication were used. In each rep, how many plants cultured?

I am not sure whether you made the nano fertilizer yourself or purchased it ready-made. In either case, the relevant information should be stated here.

Line: 252: cull fruit yield: what means?

Line 314: all abbreviations should be explained at the end of table.

All data in table 1 should be with meaning letter.

The quality of some figs is not good and data is not clear.

Line 416: I didn’t find this table and data.

Line 451: nano-NPK fertilization at 80 and 120 kg-1???

Results are primarily agronomic and economic; physiological and biochemical mechanisms of nano-NPK and SA interactions were not deeply explored (e.g., nutrient uptake kinetics, stress physiology markers).

Interaction effects (nano-NPK × SA) were mostly non-significant, yet the discussion sometimes implies strong synergy. This could be overstated.

Multiple comparisons rely heavily on ANOVA and regression, but no multivariate models (e.g., mixed models across years) were used, which might provide deeper insights.

Some economic and energy data are buried in supplementary tables (S1–S8), making it less accessible for readers who want quick comparisons.
