# Supplementary material for: Improving tomato yield and production economics with Nano-NPK fertilization and salicylic acid chemigation
Source: PLoS One. 2026 May 4;21(5):e0348207. doi: 10.1371/journal.pone.0348207 (PMC13138637; doi:10.1371/journal.pone.0348207)
Supplement: Attachment — Submitted filename: Response to Reviewers.docx [file pone.0348207.s005.docx]

**Reviewer’s comments and authors response**

We sincerely appreciate the insightful comments and valuable suggestions provided by the reviewers, particularly Reviewer 1, and the academic editor. Their constructive feedback has been instrumental in significantly enhancing the quality of our manuscript. In response, we have carefully addressed all comments, incorporated the suggested changes, added relevant references, and totally revised the manuscript to improve its clarity and scientific rigor. We are grateful for their contributions, which greatly helped us strengthen the overall quality of our work.

**General comments**

**Comment 1**. In the abstract, specify which type of NPK fertilizer was used.

**Response 1:** We applied Anderson’s Triple 20 (N 20 : P 20 : K 20), a water‑soluble chemical fertilizer suitable for growing vegetables using drip irrigation (<https://www.andersonsinc.com/>).

**Comment 2:** Keywords should be arranged alphabetically.

**Response 2:** The keywords were arranged appropriately as suggested.

**Comment 3**: Line 68: the end of paragraph you should use reference.

**Response 3**: The reference suggested by reviewers was added. Meysam Cheraghi, Babak Motesharezadeh, Hossein Ali Alikhani & Seyed Majid Mousavi (2023) Optimal management of plant nutrition in tomato (*Lycopersicon esculent* Mill) by using biologic, organic and inorganic fertilizers, Journal of Plant Nutrition 46:8, 1560-1579, DOI: 10.1080/01904167.2022.2092511

**Comment 4:** Line 148: for soil characteristics you used reference, while you should do all experiment about it. It should be mentioned how these parameters measured?

**Response 4:** Analytical procedures for both soil and irrigation water were added to the Methods and Materials section under a separate sub-heading.

A separate paper examining the impact of management systems on soil health is currently being drafted, using comprehensive information of soil and water data, and weather information.

**Comment 5:** Line 154: all characteristics of NPK need. Please write where NPK purchased? Which brand? And all-important characteristics.

**Response 5:** Andersons Triple 19 is a granular fertilizer containing 19% nitrogen, 19% phosphate, and 19% potash. It is commonly used as a starter or booster fertilizer during field preparation, just before planting vegetables and other crops (<https://www.andersonsinc.com/>).

Andersons Triple 20 is a premium, water-soluble fertilizer with 20% nitrogen, 20% phosphate, and 20% potash. It is typically applied to vegetables and other high-value crops through drip irrigation or foliar feeding. (<https://www.andersonsinc.com/>).

**Comment 6:** Line 165: please write more about the condition of growth chamber like temp, RH, Light and …

**Response 6:** The growth chamber conditions were maintained as temperatures of 26 ± 2°C for seed germination and 22 ± 2°C for early seedling growth; relative humidity at 80 ± 2% and 60 ± 2%, respectively; and light conditions of darkness for germination and 250 ± 5 µmol m⁻² s⁻¹ photosynthetic photon flux density for early seedling growth.

**Comment 7:** Line 186: according which reference?

**Response 7:** The reference already cited as 15 [Rahman, A., Thomas Harker, Wayne Lewis, KR Islam (2023) Nano and chelated iron fertilization influences marketable yield, phytochemical properties, and antioxidant capacity of tomatoes. PLOS ONE. https://doi.org/10.1371/journal.pone.0294033].

**Comment 8:** In this experiment 4 replications were used. In each rep, how many plants cultured?

**Response 8:** Ten plants per replicated plot were cultured.

**Comment 9:** I am not sure whether you made the nano fertilizer yourself or purchased it ready-made. In either case, the relevant information should be stated here.

**Response 9:** We did not synthesize any nano-fertilizers for this study. Instead, we used Nano Pro Solution^®^, a commercial product comprising nano-humic acid and nano-mesoporous silicic acid mixture, purchased from Aqua-Yield, Inc., Draper, Utah, USA (<https://www.nano-yield.com/AquaYield>). Aqua-Yield is an agri-technology company specializing in patented nano-liquid technology for agriculture.

During the tomato growing season, we applied the mixture of Nano Pro Solution through drip irrigation in combination with varying rates of water-soluble Triple 20 fertilizer at equivalent rates of N20-P20-K20, N60-P60-K60, and N100-P100-K100 kg/ha, respectively. For simplicity and clarity, we refer to this nano-fortified NPK fertilization approach as **nano-NPK** (Rahman et al., 2025; cited in the text and listed as reference 15).

**Comment 10:** Line: 252: cull fruit yield: what means?

**Response 10:** Cull fruit yield refers to the amount or number of imperfect or undesirable fruits during sorting and grading after harvest. Fruits are culled due to deformation, uneven color, damage, poor size, and disease.

**Comment 11:** Line 314: all abbreviations should be explained at the end of table.

**Response 11:** Explained accordingly.

**Comment 12:** All data in table 1 should be with meaning letter.

**Response 12:** All the mean values presented in Table 1, corresponding to the main effects of the independent variables, were denoted using different letters alphabets. As suggested, the meaning of these letters or alphabets was explained in a note at the bottom of the table. However, all the interactive effects among the factors were reported with their actual p-values to provide readers with a clearer understanding of the results.

**Comment 12:** The quality of some figs is not good and data is not clear.

**Response 1:** All the figures were enlarged and saved in higher resolution.

**Comment 13:** Line 416: I didn’t find this table and data.

**Response 13**: Due to the large volume of data and calculations, detailed information was provided in Supplementary Tables S1–S8. However, the previously presented figures have been removed, and the key economic, environmental, and greenhouse gas emission results from Tables S1–S8 are now summarized in the newly added Tables 2 and 3 for clarity.

**Comment 12**: Line 451: nano-NPK fertilization at 80 and 120 kg-1???

**Response 12**: The unit should be kg^-1^ ha^-1^ and was corrected accordingly.

**Comment 13:** Results are primarily agronomic and economic; physiological and biochemical mechanisms of nano-NPK and SA interactions were not deeply explored (e.g., nutrient uptake kinetics, stress physiology markers).

**Response 13:** Our USDA-ODA funded study was conducted is part of a multi-tiered research effort with the overreaching goal is to enhance tomato yield and fruit quality, reduce fertilizer inputs, and improve farm profitability and sustainability under Ohio’s seasonal drought conditions. Accordingly, we have collected and analyzed extensive data on tomato vegetative growth and yield attributes, nutrient and water use efficiency, phytochemical properties, antioxidant capacity, nutrient density, and environmental factors such as weather, soil, and water quality.

We would like to highlight that we published one paper, and are writing several manuscripts, covering the following key aspects of our research:

1. Enhancing tomato phytochemicals and fruit quality through nano-NPK and salicylic acid chemigation (***The paper already published in Scientific Horticulture***)
2. Tomato vegetative yield attributes, nutrient- and water use efficiency, economics, energy-use and environmental sustainability (***the current paper***)
3. Biochemical and physical aspects and nutrient content and flow dynamics.
4. Weather influences, soil health, and water quality parameters

**Comment 14:** Interaction effects (nano-NPK × SA) were mostly non-significant, yet the discussion sometimes implies strong synergy. This could be overstated.

**Response 14:** We appreciate the reviewer’s observation. While not all interaction effects between nano-NPK and SA were statistically significant, the discussion has been revised to focus primarily on the main effects of each factor. Mentions of non-significant combined effects have been moderated to avoid overstating potential synergy. The text now clarifies that the effect of nano-NPK was generally consistent or slightly enhanced regardless of the SA concentration applied, and vice versa.

**Comment 15:** Multiple comparisons rely heavily on ANOVA and regression, but no multivariate models (e.g., mixed models across years) were used, which might provide deeper insights.

**Response 15:** Data analyses were initially performed using a three-factor factorial arrangement (Nano-NPK, SA, and year) in a randomized complete block design. Based on methodological considerations, the General Linear Model (GLM) procedure in Statistical Analysis Software^®^ (SAS) was identified as the most appropriate approach for evaluating the effects of the predictor variables on dependent variables. Subsequently, a two-way analysis of variance (ANOVA) based on a linear mixed model was employed to assess the main and interactive effects of nano-NPK fertilization and SA levels (fixed variables), with block treated as a random effect across years. Data from the 2022 and 2023 growing seasons were analyzed; however, because interactions between the fixed factors and year were inconsistent, year was treated as a repeated (replication) effect. Mean comparisons for the response variables (tomato traits and economic parameters) were conducted using least square means, and differences were determined using Duncan’s Multiple Range Test (DMRT) at a significance level of α = 0.05.

**Comment 16:** Some economic and energy data are buried in supplementary tables (S1–S8), making it less accessible for readers who want quick comparisons.

**Response 16:** Due to the large volume of data and calculations, comprehensive information is presented in Supplementary Tables S1 to S8. However, a summary of the results from Tables S1 to S8 is provided in the newly added Table 2.

**Reviewers’ Specific Comments to the Author**
Please use the space provided to explain your answers to the questions above. You may also include additional comments for the author, including concerns about dual publication, research ethics, or publication ethics. (Please upload your review as an attachment if it exceeds 20,000 characters)

**Reviewer #1: Abstract**
1- Statistical method using in the study has to be stated in the Abstract section;

**Response**: Statistical method was added as “Multivariate statistical analyses”

2- What were the indices for calculating the marketable yield?

**Response:** Marketable yield was calculated as the difference between total yield minus cull yield (*Ref. # 28 in the text and in the reference list as Rahman MA, Harker T, Lewis W, Islam KR. Nano and chelated iron fertilization influences marketable yield, phytochemical properties, and antioxidant capacity of tomatoes. PLoS ONE. 2023; 18: e0294033. https://doi.org/10.1371/journal.pone.0294033*).

**Introduction**
For optimal management of plant nutrition in tomato, is needed to present some literature reviews, for example for the second paragraph of the Introduction section "lines 69-77", you may use the following work for the literature reviews:
• Optimal management of plant nutrition in tomato (Lycopersicon esculent Mill) by using biologic, organic and inorganic fertilizers. Journal of Plant Nutrition, 46(8): 1560-1579. [https://doi.org/10.1080/01904167.2022.2092511](https://urldefense.com/v3/__https:/doi.org/10.1080/01904167.2022.2092511__;!!KGKeukY!31fRhVq_d_RHcXWAdHNy2l8VzIlIwXs4_-fzuukuW-XXgd_z4ksI-HjW5ESDL5VFo1PUNTPCGQm3mfTCTQ$).

**Response:** The suggested reference “Cheraghi et al., (2023) was added accordingly.

For the lines "103-111", about the importance, functions and critical points to use Salicylic Acid in agriculture, there are some relevant and more up-to-date works to improve the literature reviews of this section; two samples are listed as follows:
1. Effect of Foliar Application of Silicon and Salicylic Acid on Regulation of Yield and Nutritional Responses of Greenhouse Cucumber Under High Temperature. Journal of Plant Growth Regulation, 41(5): 1978-1988. [https://doi.org/10.1007/s00344-021-10562-5](https://urldefense.com/v3/__https:/doi.org/10.1007/s00344-021-10562-5__;!!KGKeukY!31fRhVq_d_RHcXWAdHNy2l8VzIlIwXs4_-fzuukuW-XXgd_z4ksI-HjW5ESDL5VFo1PUNTPCGQntrh9LRA$)
2. Salicylic Acid and Biochar-Biofertilizer Improve Soil Fertility, Drought Tolerance, and Fig Yield in a Semi-Arid Region. Journal of Soil Science and Plant Nutrition, pp.1-15. [https://doi.org/10.1007/s42729-025-02609-3](https://urldefense.com/v3/__https:/doi.org/10.1007/s42729-025-02609-3__;!!KGKeukY!31fRhVq_d_RHcXWAdHNy2l8VzIlIwXs4_-fzuukuW-XXgd_z4ksI-HjW5ESDL5VFo1PUNTPCGQkknf7rmw$).

**Response:** The suggested references “Tadayon et al. (2025) and Basirat & Mousavi 2022)” were added accordingly.

The last paragraph of the introduction section, you used the term of "low fertility soils", while this term was not mentioned in the abstract, in the title, please clarify, what it does mean and it was based on which indices;

**Response:** The indices of our low fertility “Doles silt loam” soil are nutrient imbalance and deficiencies, low organic matter and nitrogen contents, low cation exchange capacity, poor soil structure with subsoil fragipan, and low microbial activity, all of which reduce its ability to support healthy plant growth.

**Materials and Methods**
In the manuscript you used "low fertility soils" but the needed information to support this term was missed in the Materials and Methods section;
**Response**: The low fertility soil term was added under the description of “Study Site”.

The software using for statistical analysis has no mention in the Materials and Methods section;
**Response**: The software used for statistical analysis included Statistical Analysis Software^®^ (SAS 9.4) and SigmaPlot^®^ 15, as described in the Materials and Methods section.

The cultivar has to be stated in the manuscript;
**Response:** The tomato cultivar name is included twice in the Abstract (line 35) and Materials and Methods section under Experimental design and cultural practices (line 169)

**Results**
Please use the figures and images with higher resolution;
**Response:** The new figures were enlarged and saved in higher resolution for better display.

**Conclusion**
What is the applied conclusion of this research for farmers and experts? Please state this matter in the conclusion section

**Response:** An applied aspect of the conclusions was added.

**Reviewer #2:**

The study’s integration of agronomic, economic, energy, and environmental assessments provides a holistic perspective on sustainable tomato production.
While the manuscript is scientifically sound and offers valuable insights, several aspects could be improved to strengthen clarity, rigor, and broader applicability.

The revised manuscript demonstrates enhanced clarity, rigor, and broader applicability, reflecting the constructive feedback from reviewers. Key improvements include a revised statistical analysis, strengthened results and discussion sections, the inclusion of recent and relevant references, and the addition of high-resolution, large-format figures. Furthermore, the conclusions have been refined to emphasize practical implications and potential applications across a wider range of contexts.
